# Supplementary material for: Coupled atmosphere-ice-ocean dynamics during Heinrich Stadial 2
Source: Nat Commun. 2022 Oct 4;13:5867. doi: 10.1038/s41467-022-33583-4 (PMC9532435; doi:10.1038/s41467-022-33583-4)
Supplement: Supplementary file 1 — Supplementary Information [file 41467_2022_33583_MOESM1_ESM.pdf]

Supplementary Information for

Coupled atmosphere-ice-ocean dynamics during Heinrich Stadial 2

Xiyu Dong[1], Gayatri Kathayat[1]*, Sune O. Rasmussen[2], Anders Svensson[2], Jeffrey P. Severinghaus[3], Hanying Li[1], Ashish Sinha[1,4], Yao Xu[1], Haiwei Zhang[1], Zhengguo Shi[1,5,6], Yanjun Cai[1], Carlos Pérez-Mejías [1], Jonathan Baker[1], Jingyao Zhao[1], Christoph Spötl[7], Andrea Columbu[8], Youfeng Ning[1], Nicolás M. Stríkis[9], Shitao Chen[10-12], Xianfeng Wang[13], Anil K. Gupta[14], Som Dutt[15], Fan Zhang[1], Francisco W. Cruz[16], Zhisheng An[5], R. Lawrence Edwards[17], Hai Cheng[1,5,18]*

[1] Institute of Global Environmental Change, Xi'an Jiaotong University, Xi’an, 710049, China;

[2] Physics of Ice, Climate and Earth, Niels Bohr Institute, University of Copenhagen, Copenhagen 2100, Denmark;

[3] Scripps Institution of Oceanography, University of California San Diego, La Jolla, CA92093, USA;

[4] Department of Earth Science, California State University, Carson, CA 90747, USA;

[5] State Key Laboratory of Loess and Quaternary Geology, Institute of Earth Environment, Chinese Academy of Sciences, Xi’an, 710061, China;

[6] Center for Excellence in Quaternary Science and Global Change, Chinese Academy of Sciences, Xi’an 710061, China;

[7] Institute of Geology, University of Innsbruck, 6020 Innsbruck, Austria;

[8] Department of Earth Sciences, University of Pisa, Via Santa Maria 53, 56126 Pisa (PI), Italy;

[9] Department of Geochemistry, Universidade Federal Fluminense, Niterói, 24020-141, Brazil;

[10] School of Geography, Nanjing Normal University, Nanjing, 210023, China;

[11] Key Laboratory of Virtual Geographic Environment (Nanjing Normal University), Ministry of Education, Nanjing, 210023, China;

[12] Jiangsu Center for Collaborative Innovation in Geographical Information Resource Development and Application, Nanjing, 210023, China;

[13] Earth Observatory of Singapore and Asian School of the Environment, Nanyang Technological University, 639798, Singapore;

[14] Department of Geology and Geophysics, Indian Institute of Technology Kharagpur, Kharagpur, India;

[15] Wadia Institute of Himalayan Geology, Dehradun, 248001, India;

[16] Instituto de Geociências, Universidade de São Paulo, São Paulo 05508-090, Brazil;

[17] Department of Earth and Environmental Sciences, University of Minnesota, Minneapolis, MN 55455, USA;

[18] Key Laboratory of Karst Dynamics, MLR, Institute of Karst Geology, CAGS, Guilin, 541004, China.

***Corresponding authors:** kathayat@xjtu.edu.cn (G.K); cheng021@xjtu.edu.cn (H.C.).

This file includes:

Supplementary Notes 1 to 11
Supplementary Tables 1 to 4
Supplementary Figures 1 to 19
Descriptions for Supplementary Datasets 1 to 3
Supplementary References

Other supplementary materials for this manuscript include the following:

Datasets 1 to 3
Supplementary Code

Supplementary Note

1.1 Composite record

As shown in Fig. 1, the $\delta^{18}O$ values of the slower growing speleothem Cherrapunji-2017-1 is systematically higher by 0.6‰ than the faster growing Cherrapunji-2, thus the Cherrapunji-2017-1 record is adjusted accordingly (Supplementary Data 2). Noticeably, a slight offset remains at 24.3–24.0 ky BP (Fig. 1). This difference stems from the higher $\delta^{18}O$ values of Cherrapunji-2017-1 during this extremely slow growth period (~0.009 mm/year) (Supplementary Fig. 4b), which is commonly observed in caves[1-3]. Over the contemporary growth interval, we exclusively use the Cherrapunji-2 record because of its more precise chronology (more ^{230}Th dates and better developed laminae) and higher resolution (Supplementary Figs. 3 and 4). By this approach, we constructed the composite Cherrapunji record (Fig. 2a and Supplementary Data 2).

1.2 Counting results and uncertainties

The counting was conducted five times for Cherrapunji-2: twice by a machine-assisted method ("Image J" and "MATLAB" software, see ref. [4]) and three times manually. The five-time counting for Cherrapunji-2017-1 was manually done. Supplementary Figure 3 shows the counting result for stage III, whose uncertainty is the 2σ result of 5x counting. The annual lamina thickness of Cherrapunji-2 obtained by the machine-assisted method is shown in Fig. 1b and Supplementary Fig. 4a. The laminae in Cherrapunji-2 are distinct with an average thickness larger than Cherrapunji-2017-1 by a factor of 5 (Fig. 1b). Hence, the lamina counting uncertainty of Cherrapunji-2 is much smaller. The majority of laminae in Cherrapunji-2 is distinct as shown in Supplementary Fig. 3c. It is noteworthy that the Asian Heinrich Period 2 (AHP2) onset is continuous in speleothem Cherrapunji-2017-1 (Supplementary Fig. 3a), whereas it is interrupted by a hiatus in Cherrapunji-2 (Supplementary Fig. 3b). Therefore, the duration of this excursion was defined by the Cherrapunji-2017-1 record instead of using the Cherrapunji composite record.

1.3 Speleothem $\delta^{18}O$ interpretations

Earlier studies have revealed apparent discrepancies in the $\delta^{18}O$ interpretation of speleothems in the Asian summer monsoon (ASM) regions at millennial-to-orbital timescales[3,5-7]. For example, Yuan[8] suggested that changes in the isotopic fractionation of water vapor along the moisture trajectory between tropical ocean sources and the cave site could explain the speleothem $\delta^{18}O$ variations. Cheng[6] suggested that changes in the annual proportion of the lighter $\delta^{18}O$ monsoon rainfall (essentially summer rainfall) could explain the speleothem $\delta^{18}O$ variations. Results from the numerical climate model[7] showed that during the Heinrich events, the higher speleothem $\delta^{18}O$ values in the East Asian summer monsoon (EASM) domain reflect the upstream transportation of isotopically enriched water vapor, which also corresponds to the weakening of the Indian summer monsoon (ISM). The climate models suggest that

the speleothem δ^{18}O reflects spatially integrated monsoon rainfall between tropical ocean sources and cave sites, which further confirms, rather than contradicts, the speleothem proxy interpretation of Cheng[6] and Yuan[8]. Recent advances in the fields of modern precipitation isotopes and climate model simulations together with the proxy studies have suggested a broad consensus that at millennial-to-orbital timescales speleothem δ^{18}O in the ASM domain reflects the large-scale monsoonal circulation controlled by moisture sources and changes driven by the overall monsoonal circulation patterns, which are independent of the cave location, and precipitation amount at the site[9-14].

1.4 Replication test

The "replication test"[3,15] for the ISM domain was conducted using Cherrapunji-2 and Cherrapunji-2017-1, and shows that the two records share an overall similarity for the majority of their contemporary sections (Supplementary Fig. 6b). Moreover, Mawmluh Cave (25°15′N, 91°42′E) is ~50 km away from Cherrapunji Cave (25°12′N, 92°27′E), and the structure of Cherrapunji and MWS-1 δ^{18}O records also replicate well (Supplementary Fig. 6d). Notably, Wulu Cave and Dongqinghe Cave are located at the transitional zone between ISM and EASM domains (Supplementary Fig. 7), so one would expect to see the structures of Wulu and Dongqinghe cave δ^{18}O records during AHP2 to some extent similar to the Cherrapunji record. Indeed, speleothem records from the Wulu and Dongqinghe caves exhibit a similar structure as the Cherrapunji record (Supplementary Fig. 7), providing a robust replication test in terms of the isotopic pattern. The Marota (MAG) and Paixão (PX-07) Cave speleothem δ^{18}O records from the NE South American summer monsoon (SASM) domain replicate with each other (Supplementary Fig. 6e). The BTV-4C record also replicate the previously published BTV-4C record (Supplementary Fig. 6f). The δ^{18}O measurements for YX-51 and PX-07 show slightly higher values (0.5‰ for YX-51 and 0.8‰ for PX-07) compared to the previous measurements (Supplementary Fig. 6a, c), although their patterns are similar. This might be because that the δ^{18}O measurements for YX-51 and PX-07 were not conducted at the center of these speleothems (Supplementary Fig. 5a, b), which possibly gives rise to some fractionation effects. The analyses at different laboratories might also contribute to small biases, i.e., MAT253 (this study), Delta Plus (previous PX-07, University of São Paulo, Brazil) and MAT253 (previous YX-51, Nanjing Normal University, China). In summary, the comparison between the δ^{18}O records of the same and different caves from the same climatic region (Supplementary Fig. 6) suggests that the speleothem δ^{18}O records broadly replicate although there are minor differences in their absolute values.

1.5 Greenland ice-core records

The Greenland ice-core δ^{18}O records utilized in this study include NGRIP, GISP2, GRIP and NEEM records[16-21]. Greenland ice-core [Ca^{2+}] records include NGRIP, GISP2, GRIP and NEEM records[20,22-24]. We refer to the Greenland ice-core records (δ^{18}O and [Ca^{2+}]) on the GICC05 chronology[20,25-27] with ages

reported as thousands of years before 1950 CE (ky BP) or years before 1950 CE (y BP). We take the reported "Maximum Counting Error" as 2σ (95%) age uncertainty and use the latest nomenclature and timing of interstadials/stadials from ref. [20].

1.6 Climatic significance of Greenland ice-core [Ca^{2+}]

1.6.1 Dust sources

Previous studies have revealed that the bulk of dust in central Greenland during the Holocene and the glacial periods arose from particles smaller than 4 μm, because large particles are depleted during long-range transport as a result of gravitational settling[28]. As for the mineral dust deposited on central Greenland during the last glacial period, the contributors are mainly, but not exclusively, the Taklimakan and the Gobi deserts in Asia[29-32]. The further information of these deserts can be found in ref. [33]. Recent work further indicates that between Greenland Stadial (GS-) 5.1 and GS-3 (~31–23 ky BP), the Asian deserts were the main dust source regions. However, dust derived from the Sahara might have increased to some extent during GS-2 (~23–15 ky BP)[34]. To sum up, the Taklimakan and Gobi deserts, hereafter referred to as the Asian dust source regions, were the main sources of mineral dust transported to Greenland during 27–23 ky BP.

1.6.2 Westerlies-dominated atmospheric fast transportation and deposition of Asian dust

The Asian dust-veiled clouds in the upper troposphere (8–10 km above the Earth's surface) are transported more than one full circuit across the boreal region within less than two weeks by the Northern Hemisphere westerly winds[35]. The dust storm prevails in the Asian dust source regions mostly during the spring season (March to May). Modern observations indicate that at the interannual timescale, there is no lag between dust emission in the Asian dust source regions and dust deposition in Greenland. For example, the dust record (expressed as Ca^{2+} with a seasonal resolution) from snow-pits (at the NGRIP ice camp) measured for the year 1998–1999 shows prominent dust spikes during the spring-summer periods, these dust spikes are an order of magnitude higher than fall-winter periods[36]. In addition, the seasonal dust record from the snow-pits near the Greenland NEEM camp (cover 2003–2009 CE) shows an anomalous dust event for the winter of 2005 and spring 2006[37], which coincides with the strongest dust storm activity reported in the Asian dust source region between 2003–2009 CE[38,39]. Congruently, during the last glacial period, boreal westerlies were more intensified than Holocene[40], and the calculated average atmospheric residence time of [Ca^{2+}] was less than 15 days[24]. The changes in both the snow accumulation rate and the [Ca^{2+}] residence time were relatively small across stadial/interstadial transitions[24,41].

1.6.3 Discrepancy between Greenland ice-core [Ca^{2+}] and Cherrapunji $\delta^{18}O$ and possible

explanations

After this +320-year shift, the speleothem Cherrapunji $\delta^{18}O$ and Greenland ice-core $[Ca^{2+}]$ records match with each other (Fig. 3). Of note, however, are a few distinctions.

(1) The amplitude of the ASM speleothem $\delta^{18}O$ shift during the weak monsoon interval between ~26.5–25.3 ky BP is smaller than that of AHP2 (Supplementary Fig. 7a–h), despite that the two periods have a similar amplitude in Greenland ice-core $[Ca^{2+}]$ and dust records (Fig. 3b and Supplementary Fig. 12b). Based on this observation, we surmise that although the "stadial" between 26.5–25.3 ky BP is of the non-Heinrich type, its climatic impact on the meridional position/strength of the Northern Hemisphere westerly winds might be comparable to the Heinrich stadial 2. Alternatively, this may suggest an influence from other potential dust sources besides the Asian dust sources, which warrants further studies.

(2) The double-spike structure of DO-2 in the Cherrapunji $\delta^{18}O$ record is not as prominent as the counterpart in Greenland $[Ca^{2+}]$ records (Fig. 3). We contend that as the westerlies were displaced to higher latitudes during the onset of DO-2.2, Greenland ice-core $[Ca^{2+}]$ might be affected more by climatic factors of high-latitude than mid- to low-latitudes in Northern Hemisphere. The relatively smooth DO-2 structure in the Cherrapunji record may be due to the inhibited heat and moisture transport from the low latitudes to the ASM region during the Last Glacial Maximum[42]. It may be also associated with the influence of the hydrological cycle in the tropics[43].

1.7 Uncertainty due to tuning the Greenland $[Ca^{2+}]$ time-series to the Cherrapunji $\delta^{18}O$ record

We have provided a dynamic basis for the correlation between Greenland ice-core $[Ca^{2+}]$ and ASM speleothem $\delta^{18}O$ records (see main text). However, several factors must also be considered when tuning the ice-core $[Ca^{2+}]$ to Cherrapunji $\delta^{18}O$ record.

First, we argue in the Supplementary Note 1.6.2 that the Greenland ice-core $[Ca^{2+}]$ variation can be linked to the change in the Asian dust source without a considerable lag on interannual timescale. However, the relationship between Greenland $[Ca^{2+}]$ changes and the ASM speleothem $\delta^{18}O$ changes is complex on the interannual scale and may involve additional uncertainty. To obtain a conservative estimate, we assume a 20-year uncertainty ($uncertainty_1$) for it.

Second, we considered the tie point uncertainty in the Cherrapunji $\delta^{18}O$ record and the tie point uncertainty in the ice-core $[Ca^{2+}]$ record. To obtain a conservative estimate, we chose the maximum tie point uncertainty in the Cherrapunji record (60 years, $uncertainty_2$) and the maximum tie point uncertainty in the $[Ca^{2+}]$ record (60 years, $uncertainty_3$) (Supplementary Table 3) in the calculations. The tie point uncertainty of the Cherrapunji record includes both the age model uncertainty and the change point uncertainty (Supplementary Table 2).

Accordingly, after considering these possible factors that may contribute to the overall uncertainty, we quadratically combined $uncertainty_1$ (20 years), $uncertainty_2$ (60 years) and $uncertainty_3$ (60 years) and derived an overall uncertainty of 90 years (2σ). We contend that this uncertainty is a conservative estimate for tuning the Greenland [Ca^{2+}] time-series to the Cherrapunji $\delta^{18}O$ record between 27–23 ky BP.

1.8 Marine sediment records

Using a deposition modeling technique, a recent study[44] established age-depth models for 92 marine sediment cores from the Atlantic Ocean that are consistent with the Greenland GICC05 chronology. However, this chronology still bears larger uncertainties than our Cherrapunji $\delta^{18}O$ record. In view of the improvement of the GICC05 chronology in our study, we shifted these records by +320 years as well (Supplementary Fig. 15). For other published marine sediment records their chronologies remain unchanged (Supplementary Fig. 15).

1.9 Antarctic ice-core records

In our study, we have used the oxygen isotope ($\delta^{18}O$) records from WAIS Divide Ice-core (WDC)[45] and EPICA Dronning Maud Land (EDML)[46]. We have also used an averaged deuterium excess (d_{ln}) record, by averaging the d_{ln} records from WDC, EDML, Dome Fuji (DF), EPICA Dome C (EDC), and Talos Dome ice-cores (Supplementary Fig. 2)[47]. The non-sea-salt soluble calcium ([$nssCa^{2+}$]) record[48] from the WDC ice-core was also used. The Antarctic ice-core proxies used in this study are on the WD2014 chronology[49]. It is noteworthy that d_{ln} records from the EDML, DF, EDC and Talos Dome ice-cores as well as $\delta^{18}O$ record from EDML ice-core have been tuned to the WD2014 chronology in a previous study[47].

The Antarctic ice-core $\delta^{18}O$ is interpreted as a proxy for local condensation temperature[45].

In our study, we have used the logarithmic definition of ice-core deuterium-excess parameter, i.e., d_{ln}, which is a more reliable proxy for moisture source variability compared with the linear definition[50]. The 5-core averaged d_{ln} records are suggested to reflect changes in atmospheric circulation and vapor transport pathways, which are associated with the latitudinal migration of the Southern Hemisphere westerly wind[47].

The ice-core [$nssCa^{2+}$] is considered a proxy of dust, and it is commonly used to trace wind field and hydrological changes in the source regions[41,51,52]. South America is suggested to be the dominant source region for the dust transported to Antarctica[53,54]. It is argued that changes in ice-core [$nssCa^{2+}$] are linked to the latitudinal position/strength of the Southern Hemisphere westerly wind[51,55].

1.10 The previously defined uncertainty of WD2014 chronology

In this study, we propose a +400-year shift of the WD2014 chronology (both ice and gas ages; see main text), which is larger than the previously defined uncertainty (~250 years for ice age and ~270 years for gas age)[49]. Indeed, this is a plausible scenario, because the annual layer thickness of the WDC ice core decreased over the Last Glacial Maximum[49], possibly leading to an undercounting of ice layers in the WDC ice core. Moreover, as argued by ref. [49]: "*the aerosol records did not have sufficient depth resolution for reliable identification of the annual signal so the annual-layer interpretation is based solely on electrical conductivity measurement*" between the depth of 2300–2711 m (15,302–26,872 y BP), which might also create a chronological bias.

1.11 Lack of stage III in records from the EASM domain

The large excursion (stage III) is prominent in speleothems from the ISM regime (Fig. 2). However, it is not expressed in the EASM speleothem $\delta^{18}O$ records (Fig. 2 and Supplementary Fig. 7). One possible explanation is that the full glacial boundary conditions might have counteracted the further increase in precipitation $\delta^{18}O$ values ($\delta^{18}O_p$) in the EASM domain. Notably, the meridional position of the westerly wind relative to the Tibetan Plateau could influence the stepwise shift of the East Asian rainfall seasons and concomitant changes of $\delta^{18}O_p$[56-59]. During AHP2, a prolonged southward shift of the westerlies is superimposed on the condition of a maximum expansion of the East Asian continent during the Last Glacial Maximum sea-level low stand[60] (Supplementary Fig. 7). This could further prevent the low-level monsoonal flow (isotopically light) from penetrating into the interior of the EASM domain[56,61]. The large increase in EASM speleothem $\delta^{18}O$ (~1‰) at the start of the stage III indicate such changes. However, the subsequently further southward-shift of the westerly wind would not significantly change the already diminished low-level monsoonal flow in the current EASM region to facilitate the stage III excursion (Supplementary Fig. 7). In contrast, Cherrapunji Cave is located the south of the Tibetan Plateau (Supplementary Fig. 11) and is less affected by the Last Glacial Maximum conditions (e.g., sea-level drop) (Supplementary Fig. 7i). In addition, changes of moisture sources, and the Western Pacific Subtropical High, Pacific Decadal Oscillation and aerosol feedbacks could play a role to some extent, which calls for follow-up research to disentangle the underlying mechanism(s).

Supplementary Tables

Supplementary Table 1 Search intervals used for the Ramp-fitting and BREAKFIT analyses

Stages	Record	Search interval (y BP)	Algorithm used	Passed sensitivity test? Y(yes)/N(no)
**Speleothems**				
**AHP2 onset**	Cherrapunji $\delta^{18}O$	24,300–24,540	BREAKFIT	Y
**AHP2 termination**	Cherrapunji $\delta^{18}O$	23,350–24,090	Ramp-fitting	Y
**SAHP2 onset**	MAG $\delta^{18}O$	24,320–24,580	BREAKFIT	Y
	BTV-4C $\delta^{18}O$	24,030–24,630	Ramp-fitting	Y
**SAHP2 termination**	MAG $\delta^{18}O$	23,710–24,310	Ramp-fitting	Y
	BTV-4C $\delta^{18}O$	23,480–24,280	Ramp-fitting	Y
	NAR-C $\delta^{18}O$	23,300–24,210	BREAKFIT	Y
**Large excursion**	Cherrapunji $\delta^{18}O$	24,150–24,510	BREAKFIT	Y
	PX-07 $\delta^{18}O$	24,210–24,490	BREAKFIT	Y
	MAG $\delta^{18}O$	24,240–24,440	BREAKFIT	Y
	PA-LA-1 $\delta^{18}O$	24,100–24,400	BREAKFIT	Y
	Tie points in Cherrapunji $\delta^{18}O$	22,940–23,540	BREAKFIT	Y
		24,460–24,700	BREAKFIT	Y
		24,760–25,000	BREAKFIT	Y
		25,110–25,510	BREAKFIT	Y
		26,350–26,650	BREAKFIT	Y
**Greenland ice-cores**				
**Greenland Interstadial 2**	NEEM $\delta^{18}O$	23,510–23,860	Ramp-fitting	Y
	Tie points in Greenland [Ca^{2+}]	23,100–23,360	BREAKFIT	Y
		24,480–24,680	BREAKFIT	Y
		24,700–25,050	BREAKFIT	Y
		25,050–25,550	BREAKFIT	Y
		26,300–26,700	BREAKFIT	Y
**Antarctica ice-cores**				
	5-core d_{ln} anomaly	23,970–24,670	Ramp-fitting	Y
**AIM2 warming**	WDC $\delta^{18}O$	23,500–25,300	Ramp-fitting	Y
	EDML $\delta^{18}O$	23,700–25,300	Ramp-fitting	Y
**AIM2 cooling**	WDC $\delta^{18}O$	22,100–24,200	Ramp-fitting	N
	EDML $\delta^{18}O$	23,000–24,100	Ramp-fitting	N

Supplementary Table 2 Age and combined uncertainties for critical change points

Stages	Record	Age model uncertainty (years)	Change point uncertainty (years)	Age and combined uncertainty (y BP)
**Speleothems**				
**AHP2 onset**	Cherrapunji $\delta^{18}O$	40	20	24,430 ± 50
**AHP2 termination**	Cherrapunji $\delta^{18}O$	20	50	23,910 ± 50
		20	60	23,510 ± 60
**SAHP2 onset**	MAG $\delta^{18}O$	50	20	24,450 ± 50
	BTV-4C $\delta^{18}O$	80	150	24,360 ± 170
**SAHP2 termination**	MAG $\delta^{18}O$	50	100	24,130 ± 110
		40	90	23,830 ± 100
	BTV-4C $\delta^{18}O$	50	70	24,040 ± 85
		70	110	23,830 ± 130
	NAR-C $\delta^{18}O$	70	70	24,040 ± 100
**Large excursion**	Cherrapunji $\delta^{18}O$	20	55	24,320 ± 60
	PX-07 $\delta^{18}O$	30	30	24,330 ± 40
	MAG $\delta^{18}O$	60	20	24,350 ± 60
	PA-LA-1 $\delta^{18}O$	~250	30	24,230 ± 250
	Tie points in Cherrapunji $\delta^{18}O$	20	40	23,240 ± 50
		40	40	24,580 ± 60
		20	20	24,885 ± 30
		20	20	25,310 ± 30
		20	20	26,490 ± 30
**Greenland ice-cores**				
**Greenland Interstadial 2**	NEEM $\delta^{18}O$	90	100	23,670 ± 130
		90	40	23,580 ± 100
**Antarctic ice-cores**				
	5-core d_{ln} anomaly	90	85	24,430 ± 120
**AIM2 warming**	WDC $\delta^{18}O$	90	190	24,570 ± 210
		90	190	24,220 ± 210
	EDML $\delta^{18}O$	90	220	24,630 ± 240
		90	240	24,170 ± 260

The age model uncertainty is according to Supplementary Data 2, the change point uncertainty is according to BREAKFIT[62] and Ramp-fitting[63] algorithms. In cases where the plus and minus errors are asymmetrical, we take the maximum errors. The combined uncertainty is the square root of the age model uncertainty and change point uncertainty (see Methods), the age model uncertainties of Greenland and Antarctic ice-core records are according to the discussion in the main text.

Supplementary Table 3 Results of tie points between Cherrapunji $\delta^{18}O$ and Greenland ice-core [Ca^{2+}] records

Tie points in speleothem and uncertainty	Ice cores	Tie points in ice cores and uncertainty (y BP)	Average timing for tie points in ice cores (y BP)	Ice-core maximum counting uncertainty (years)
23,240 ± 50*	GRIP	23,230 ± 20**	23,210 ± 50	± 590
	NGRIP	23,210 ± 20		
	GISP2	23,210 ± 40		
	NEEM	23,190 ± 20		
24,430 ± 50	GRIP	—	~24,420[26]	± 640
	NGRIP	—		
	GISP2	—		
	NEEM	—		
24,580 ± 60	GRIP	—	24,580 ± 60	± 670
	NGRIP	24,590 ± 50		
	GISP2	24,570 ± 40		
	NEEM	—		
24,885 ± 30	GRIP	24,850 ± 20	24,850 ± 30	± 690
	NGRIP	24,850 ± 20		
	GISP2	24,850 ± 20		
	NEEM	—		
25,310 ± 30	GRIP	25,330 ± 30	25,320 ± 60	± 710
	NGRIP	25,330 ± 30		
	GISP2	—		
	NEEM	25,290 ± 50		
26,490 ± 30	GRIP	26,570 ± 20	26,520 ± 60	± 770
	NGRIP	26,490 ± 30		
	GISP2	26,510 ± 50		
	NEEM	—		

*The error is the combined uncertainty (including age model uncertainty and change point uncertainty, Supplementary Table 2), the same for the other errors in the same column.

**the tie points and associated uncertainties in Greenland ice-core records are calculated using the BREAKFIT algorithm[62].

We refer to the Greenland ice-core records on the improved chronology (GICC05 age +320 years, see main text) with all ages reported as years before 1950 CE (y BP). The uncertainties of ice-core records follow ref. [20]. All the uncertainties are 2σ.

Supplementary Table 4 Additional test on the influence of the asymmetrical age models on change point detections

**Stages**	**Records**	**X^{th} percentile age model**	**Timing of change point (y BP)**	**Average timing of change point (y BP)***	**Change point obtained via median age model (y BP)**
**AHP2 onset**	Cherrapunji $\delta^{18}O$	2.5	24,380	24,420	24,430
		97.5	24,460		
**SAHP2 onset**	MAG $\delta^{18}O$	2.5	24,400	24,440	24,450
		97.5	24,470		
	BTV-4C $\delta^{18}O$	2.5	24,240	24,360	24,360
		97.5	24,480		
**AHP2 termination**	Cherrapunji $\delta^{18}O$	2.5	23,880	23,900	23,910
		97.5	23,920		
		2.5	23,490	23,510	23,510
		97.5	23,530		
**SAHP2 termination**	MAG $\delta^{18}O$	2.5	24,110	24,170	24,130
		97.5	24,220		
		2.5	23,660	23,770	23,830
		97.5	23,880		
	BTV-4C $\delta^{18}O$	2.5	23,980	24,050	24,040
		97.5	24,110		
		2.5	23,790	23,860	23,830
		97.5	23,920		
	NAR-C $\delta^{18}O$	2.5	23,980	24,050	24,040
		97.5	24,110		

*This column shows the average timing of change point derived via 2.5^{th} and 97.5^{th} percentile age models, which is comparable to the change point derived via the median (50th) age model.

Supplementary Figures

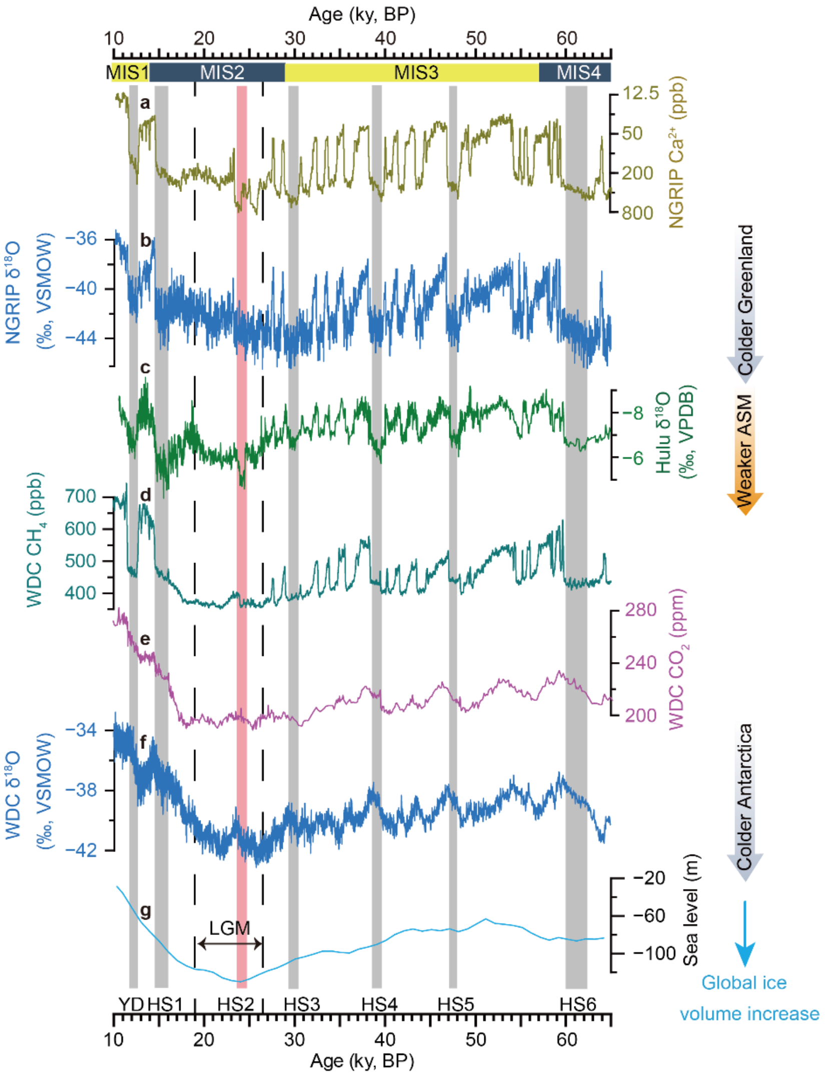

Supplementary Fig. 1 Comparison of the hydroclimate records during the late last glacial period. (**a**) Greenland NGRIP ice-core [Ca^{2+}] record (note the inverted logarithmic [Ca^{2+}] y-axis)[20]; (**b**) NGRIP ice-core $\delta^{18}O$ record[19,20]; (**c**) Speleothem $\delta^{18}O$ record from Hulu Cave (note the inverted y-axis)[64]. (**d**) Antarctica WDC ice-core CH_4 record[65], (**e**) WDC ice-core CO_2 record[66], (**f**) WDC ice-core $\delta^{18}O$ record[45] and (**g**) Global sea level stack[67]. The Marine Isotope Stages (MIS) are shown at the top. The approximate time intervals of millennial-scale events (Younger-Dryas (YD) and Heinrich Stadials (HSs)) are depicted by vertical bars and labeled at the bottom. Greenland ice-core records are plotted on the GICC05 chronology (Supplementary Note 1.5), Antarctic ice-core records are plotted on the WD2014 chronology[49,68], and the Last Glacial Maximum (LGM)[69] is depicted by a double-sided arrow. ASM: Asian summer monsoon.

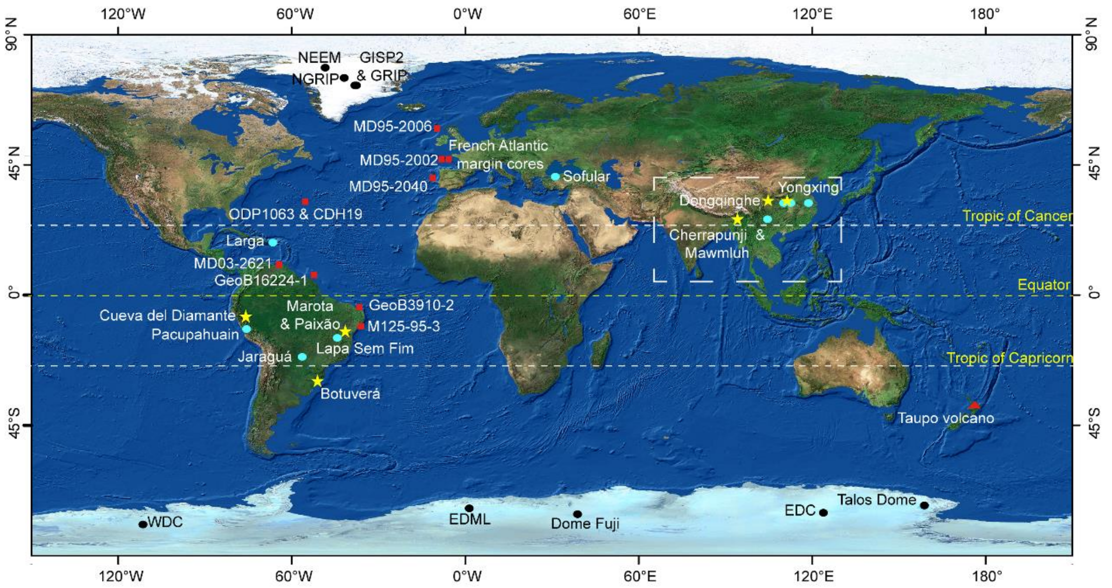

Supplementary Fig. 2 Proxy record locations. Yellow stars show locations for the caves presented in this study. Cyan dots indicate locations of other caves for comparison. Greenland and Antarctic ice-core locations are shown by black dots. Marine sediment records are delineated by red squares. Taupo volcano is depicted by a red triangle. The map was created using ArcGIS software and the world map background data are from the Environmental Systems Research Institute (ESRI, https://www.arcgis.com/apps/mapviewer/index.html?layers=10df2279f9684e4a9f6a7f08febac2a9). The enlarged section of the dotted white rectangular box is shown in Supplementary Fig. 7i. Marine records GeoB16224-1[70], M12-595-3[71] and GeoB3910-2[72] indicate an increase in rainfall in Northeast Brazil and the Amazon Basin during SAHP2, anti-phase with the Cariaco Basin (MD03-2621[73]). The termination of SAHP2 in GeoB16224-1[70], M12595-3[71] and GeoB3910-2[72] records show a decrease of rainfall in Northeast Brazil and the Amazon Basin, consistent with speleothem records. The large uncertainties (~ ±300–±700 years, 2σ) in the chronologies of marine records, however, preclude a precise correlation of the hydroclimatic changes during South American Heinrich Period 2 termination between marine and speleothem records.

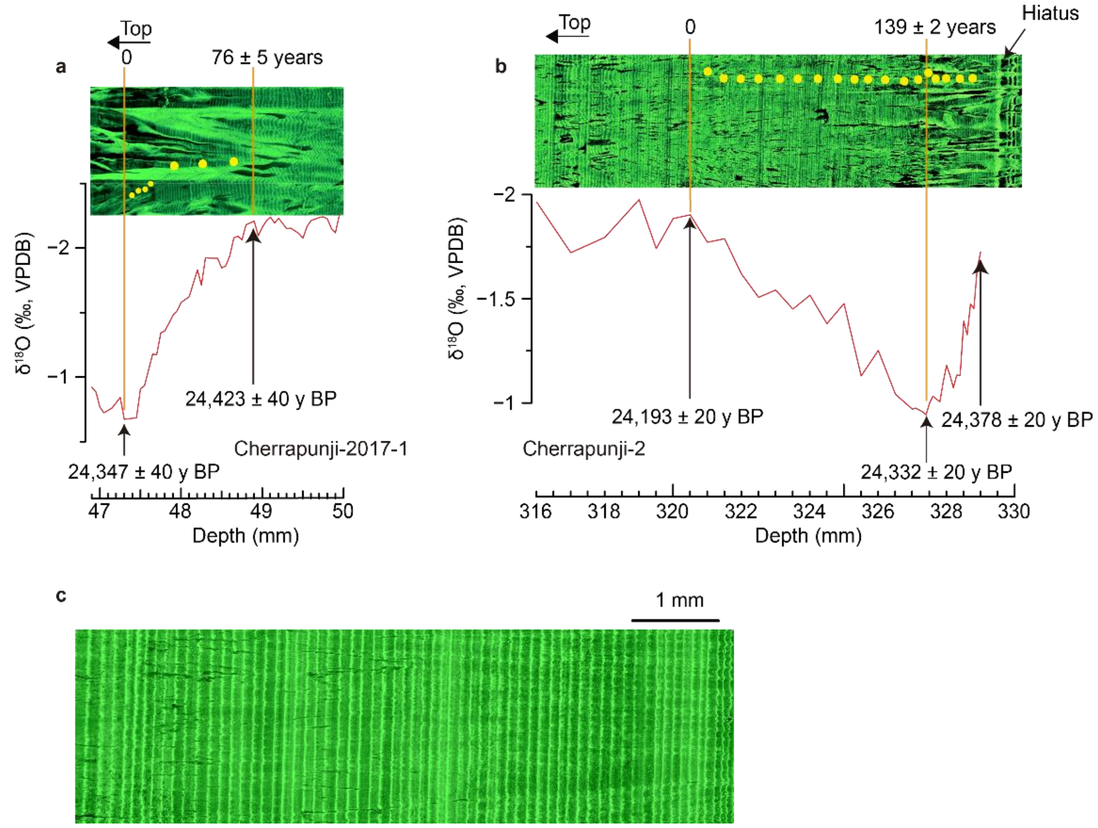

Supplementary Fig. 3 Annual lamina counting for key intervals. (**a**) Laser scanning confocal microscopy image of speleothem Cherrapunji-2017-1. Red curve is the plot of Cherrapunji-2017-1 $\delta^{18}O$ versus depth. The ages of change points and lamina counting results are shown. The laminae are counted as ten between adjacent yellow dots. (**b**) Plot of Cherrapunji-2 $\delta^{18}O$ versus depth (red curve). The labels and dots are the same as in (a). (**c**) Laser scanning confocal microscopy image of speleothem Cherrapunji-2 between 106–113 mm as an example for its clear lamination. The uncertainties of the ages of change points in (**a**) and (**b**) depict the age model uncertainty (2σ).

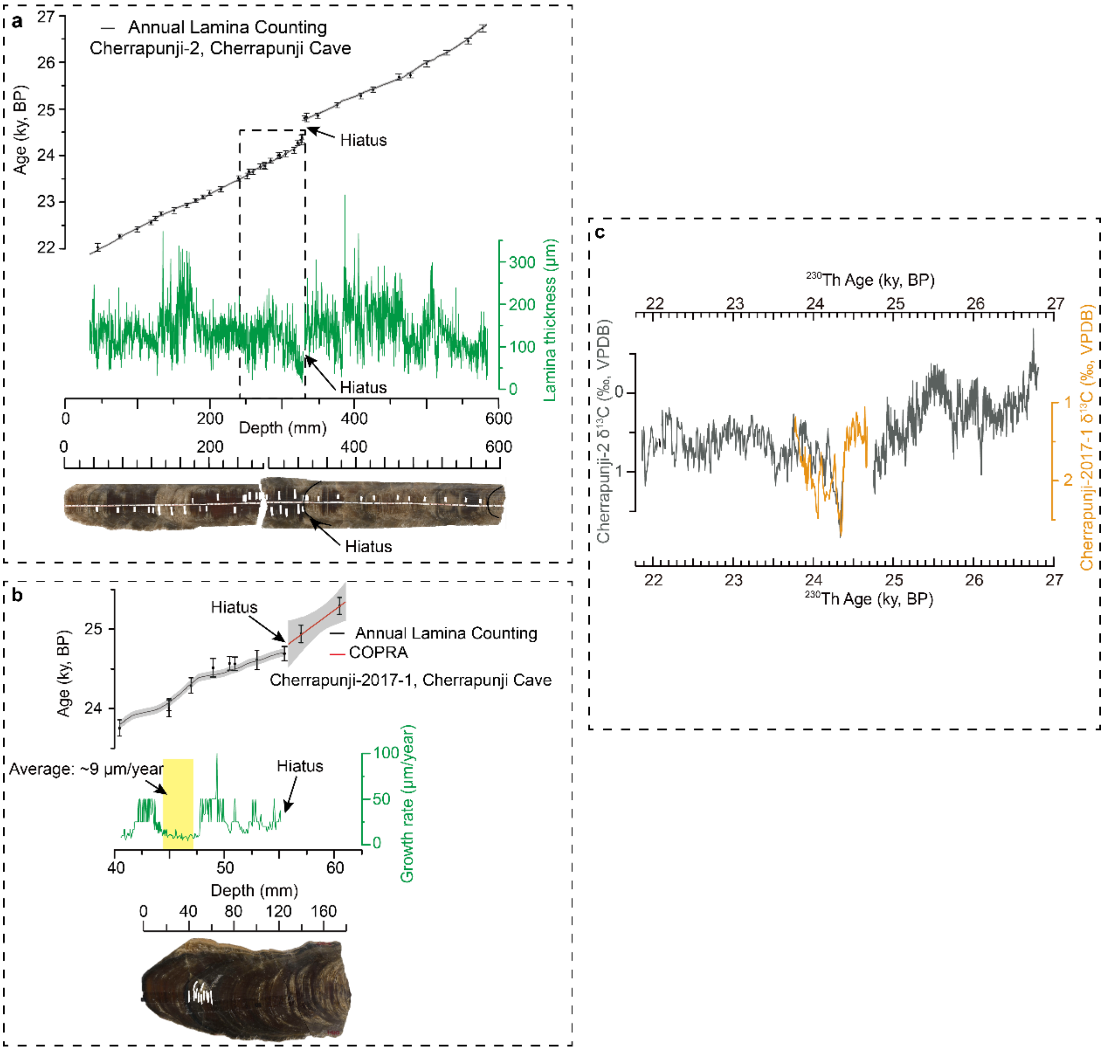

Supplementary Fig. 4 Age models and carbon isotope records of Cherrapunji Cave speleothems. (**a**) Scan images and age models of the speleothem Cherrapunji-2 (between 0–610 mm). Annual lamina thickness is shown (green curve, Supplementary Data 1). The dotted box indicates the time interval of AHP2. (**b**) Scan images and age models of the speleothem Cherrapunji-2017-1. Annual growth rate is shown (green curve), the vertical yellow bar depicts the section where the growth was extremely slow. The white bars in (**a**) and (**b**) indicate positions of subsamples for ^{230}Th dating (Supplementary Data 1). The black curves in (**a**) and (**b**) represent the mean values of the age models obtained via a combination of annual lamina counting and ^{230}Th dating. The red curve in (**b**) shows modelled mean values using the COPRA algorithm[74]. The gray bands in (**a**) and (**b**) depict the age model uncertainties (2σ). Error bars on ^{230}Th dates represent 2σ analytical error. (**c**) Comparison of the carbon isotope (δ^{13}C) records of speleothem Cherrapunji-2 and Cherreapunji-2017-1.

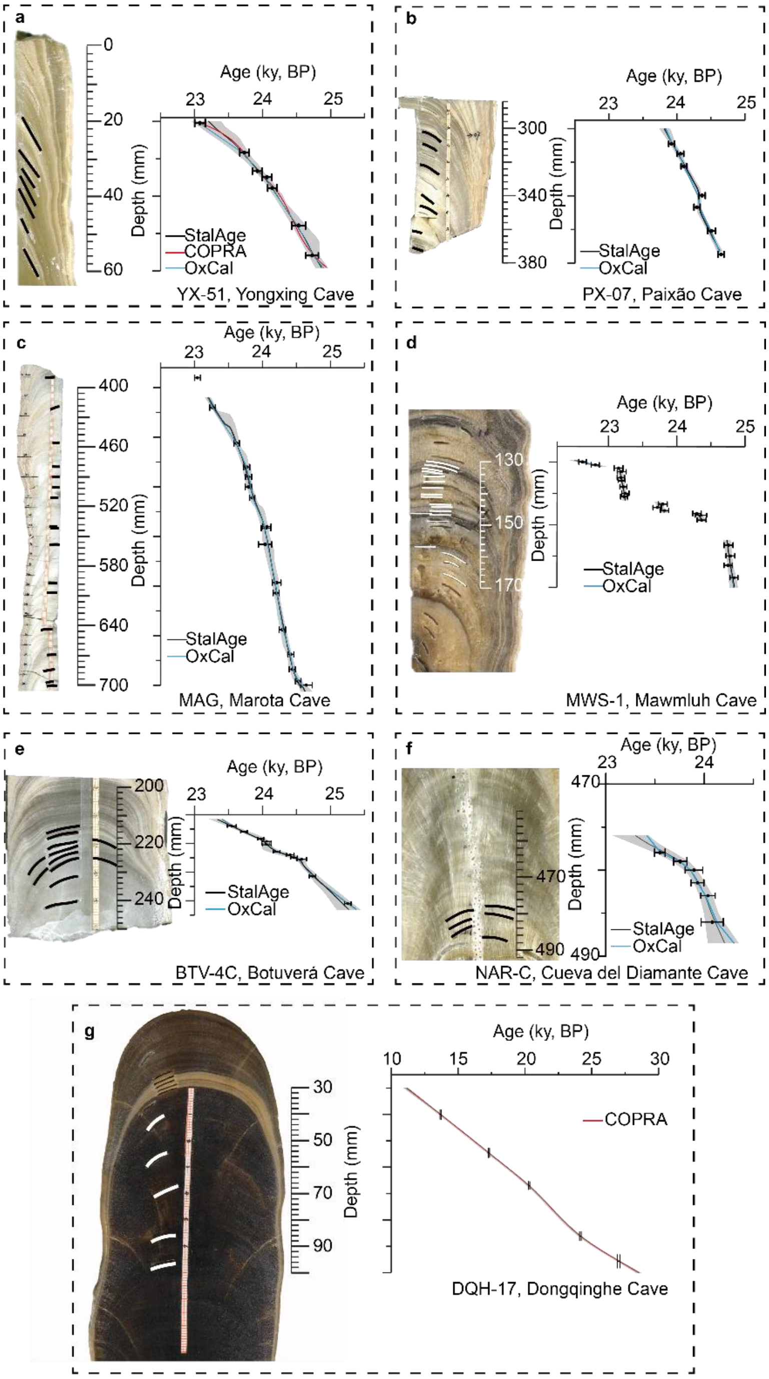

Supplementary Fig. 5 Age models and slabbed images of speleothems. (**a**)–(**g**) Scan images and age models of the speleothem YX-51 (between 0–80 mm), PX-07 (between 285–373 mm), MAG (between 390–795 mm), MWS-1 (between 115–195 mm), BTV-4C (between 200–250 mm), NAR-C (between 460–490 mm) and DQH-17 (between 0–130 mm). The white/black bars indicate positions of subsamples for ^{230}Th dating (Supplementary Data 1). The curves show modelled mean values using different age-modeling algorithms (color-coded)[74-76], the gray band depicts the 95.4% confidence interval using the StalAge[76] (**a**–**f**) and COPRA algorithms[74] (**g**). Error bars on ^{230}Th dates represent 2σ analytical error.

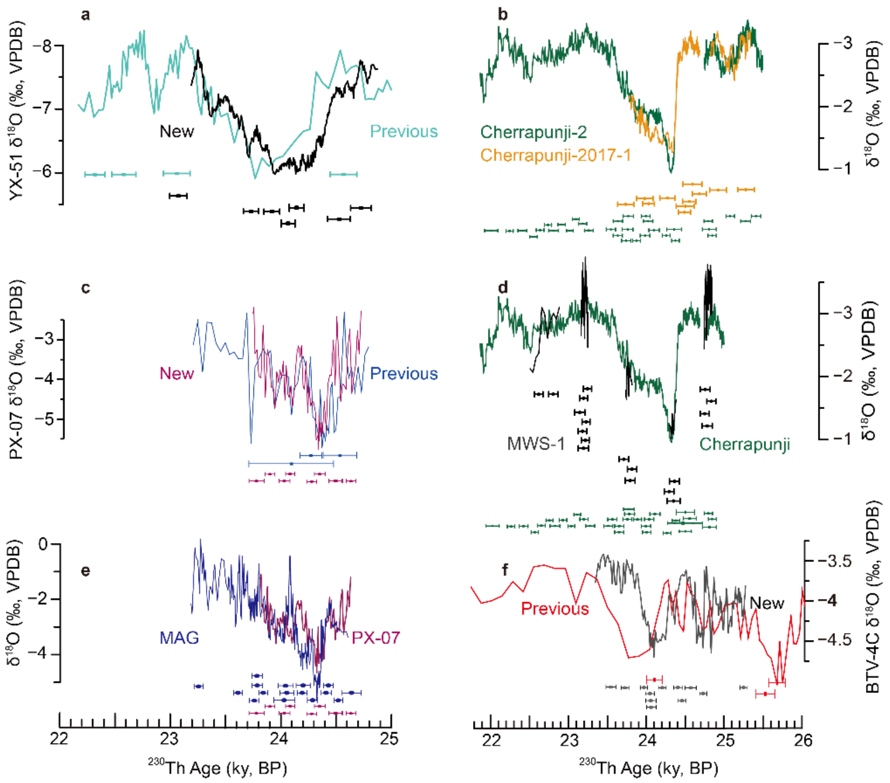

Supplementary Fig. 6 Replication test. (**a**) Replication test between $\delta^{18}O$ records of the new (black, this study) and previous[77] (cyan) speleothem YX-51 from Yongxing Cave, (**b**) replication test between $\delta^{18}O$ records of speleothem Cherrapunji-2 (green, this study) and Cherrapunji-2017-1 (mustard, this study), both from Cherrapunji Cave, (**c**) replication test between $\delta^{18}O$ records of the new (magenta, this study) and previous[78] (blue) speleothem PX-07 from Paixão Cave, (**d**) replication test between $\delta^{18}O$ records of speleothem Cherrapunji (green, composite record from Cherrapunji Cave, this study) and MWS-1 (black, Mawmluh Cave, this study), (**e**) replication test between $\delta^{18}O$ records of speleothem MAG (blue, Marota Cave, this study) and PX-07 (magenta, Paixão Cave, this study), (**f**) replication test between $\delta^{18}O$ records of the new (black, this study) and previous[79] (red) speleothem BTV-4C from Botuverá Cave. Error bars show ^{230}Th dates with uncertainties (2σ) for each record (color coded). It is noted that the $\delta^{18}O$ absolute values of the new measurements show slight offset with previous measurements in (**a**) and (**c**) (Supplementary Note 1.4), thus the new measurements were shifted by −0.5‰ (**a**) and −0.8‰ (**c**), respectively. Cherrapunji-2017-1 (**b**) and MWS-1 (**d**) $\delta^{18}O$ records were shifted by −0.6‰ and −0.5‰, respectively.

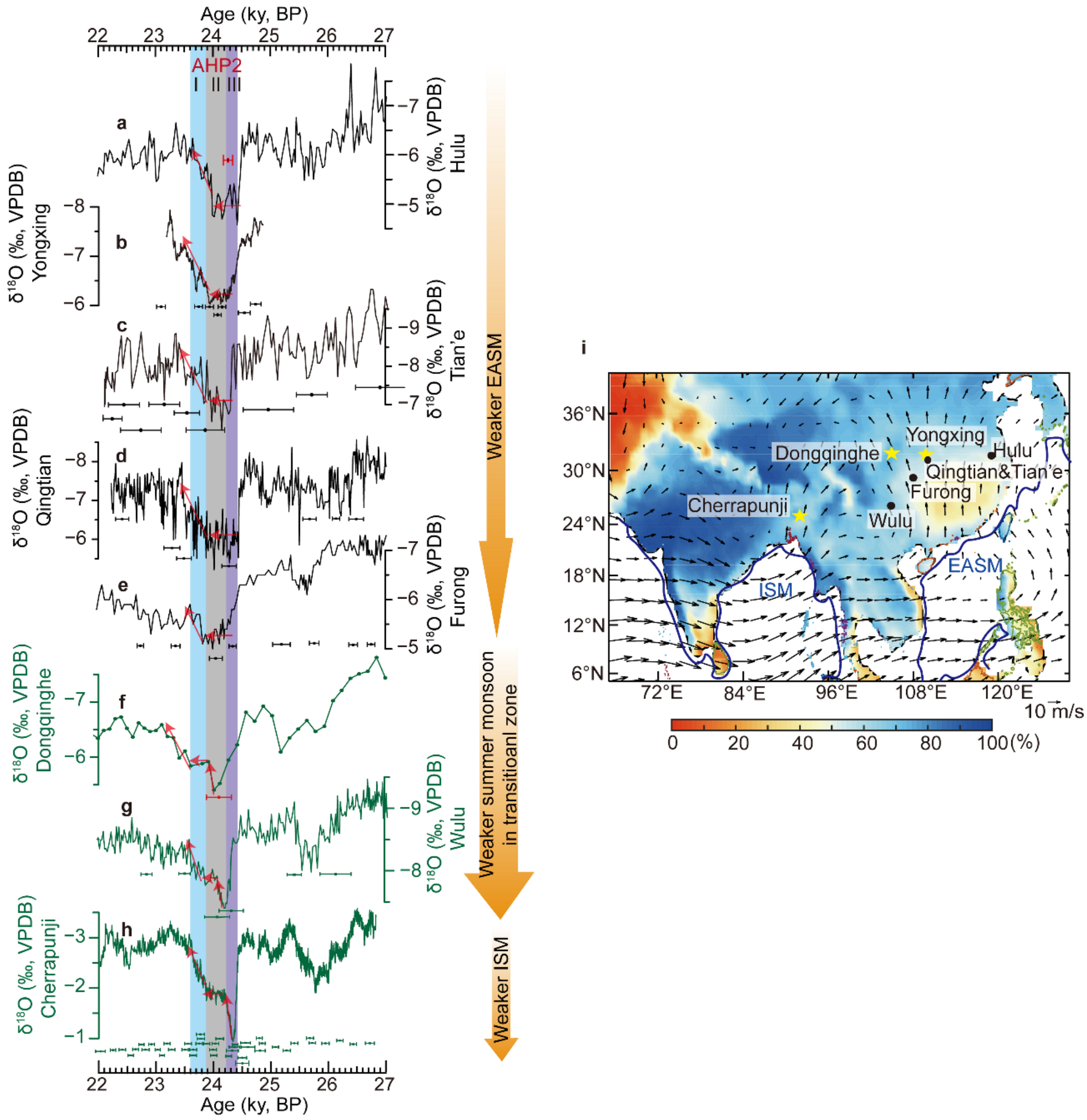

Supplementary Fig. 7 Comparison between speleothem δ¹⁸O records from the Asian summer monsoon domain. (**a**) to (**e**) are speleothem δ^{18}O records from Hulu[64], Yongxing (this study), Tian'e[80], Qingtian[81] and Furong[82] caves in the EASM domain. (**f**) and (**g**) are speleothem δ^{18}O records from Dongqinghe Cave (DQH-17, this study) and Wulu Cave[83], respectively, both from the transitional zone between the EASM and ISM domains. (**h**) Speleothem δ^{18}O record from Cherrapunji Cave (this study) in the ISM domain. Black and green error bars show ^{230}Th dates and their 2σ errors. The red error bars depicts the age model uncertainties of the Hulu Cave record[64] and Dongqinghe Cave record (this study). The red arrows indicate the trends of different stages in these records. The vertical colored bars are the same as in Fig. 2. (**i**) The background is the average percentage of June–September (JJAS) precipitation (color scale) in 1981–2010 CE from the Global Precipitation Climatology Center (GPCC)[84]. JJAS low-level wind vectors (black arrows) are based on the Modern-Era Retrospective analysis for Research and Applications database[85]. Yellow stars show locations for the caves presented in this study. Cyan dots indicate locations of other caves for comparison. The purple contour line represents the coastlines of sea level low-stands at about −120 m. ISM: Indian summer monsoon; EASM: East Asian summer monsoon.

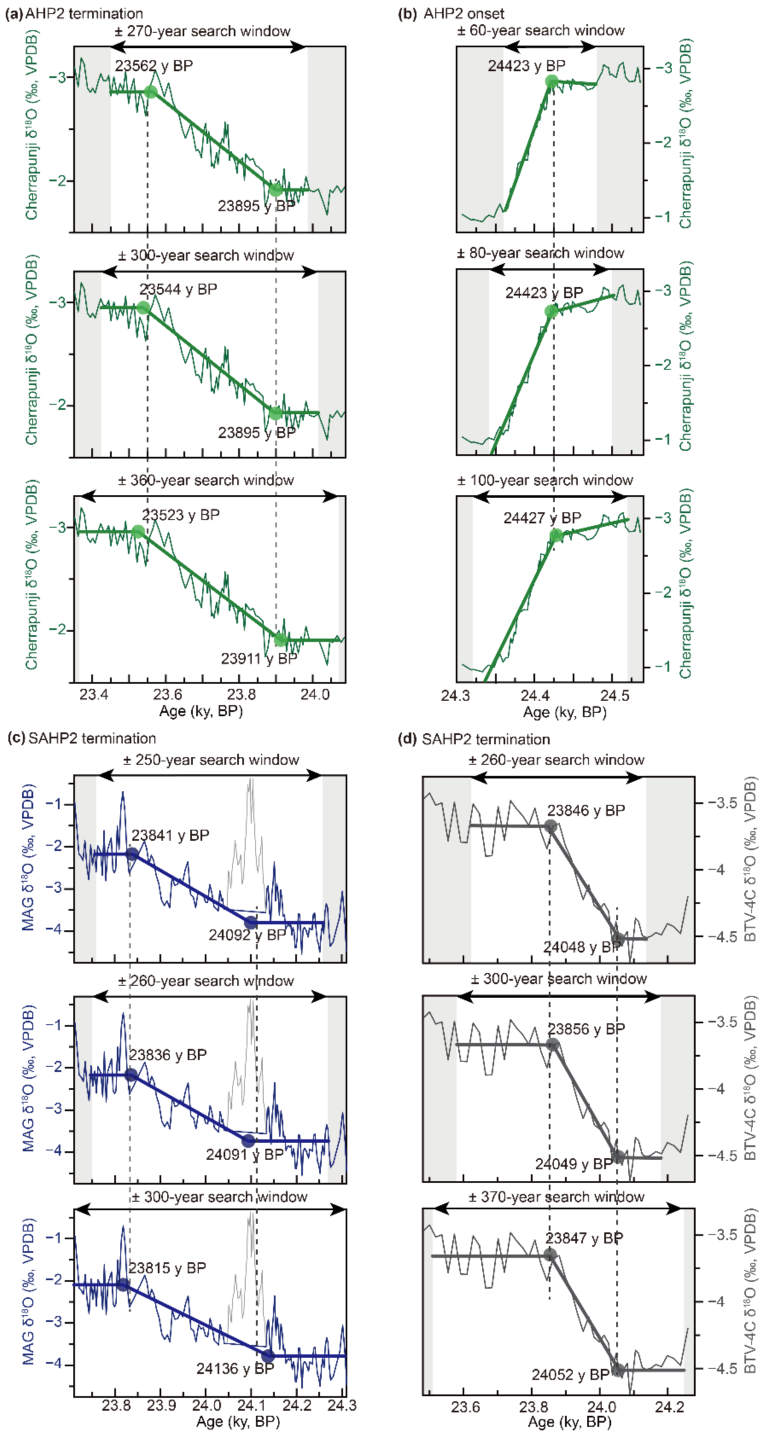

Supplementary Fig. 8 Sensitivity tests on the speleothem $\delta^{18}O$ transitions during (a) AHP2 termination, (b) AHP2 onset, (c) SAHP2 termination (MAG) and (d) SAHP2 termination (BTV-4C). The bold ramps in (**a**), (**c**) and (**d**) are defined by the Ramp-fitting algorithm[63] (see Methods) over three search windows of different widths. The bold lines in (**b**) are generated by BREAKFIT algorithm[62] over three search windows of different widths. The change points are shown and the selected search windows are indicated by the horizontal black arrows and the white boxes. The vertical dashed lines show the variation of change points when search time window changed. The calculation of SAHP2 termination in (**c**) is based on the contributory record (blue) while the large dry event was cropped (gray) (see Methods). AHP2: Asian Heinrich Period 2; SAHP2: South American Heinrich Period 2.

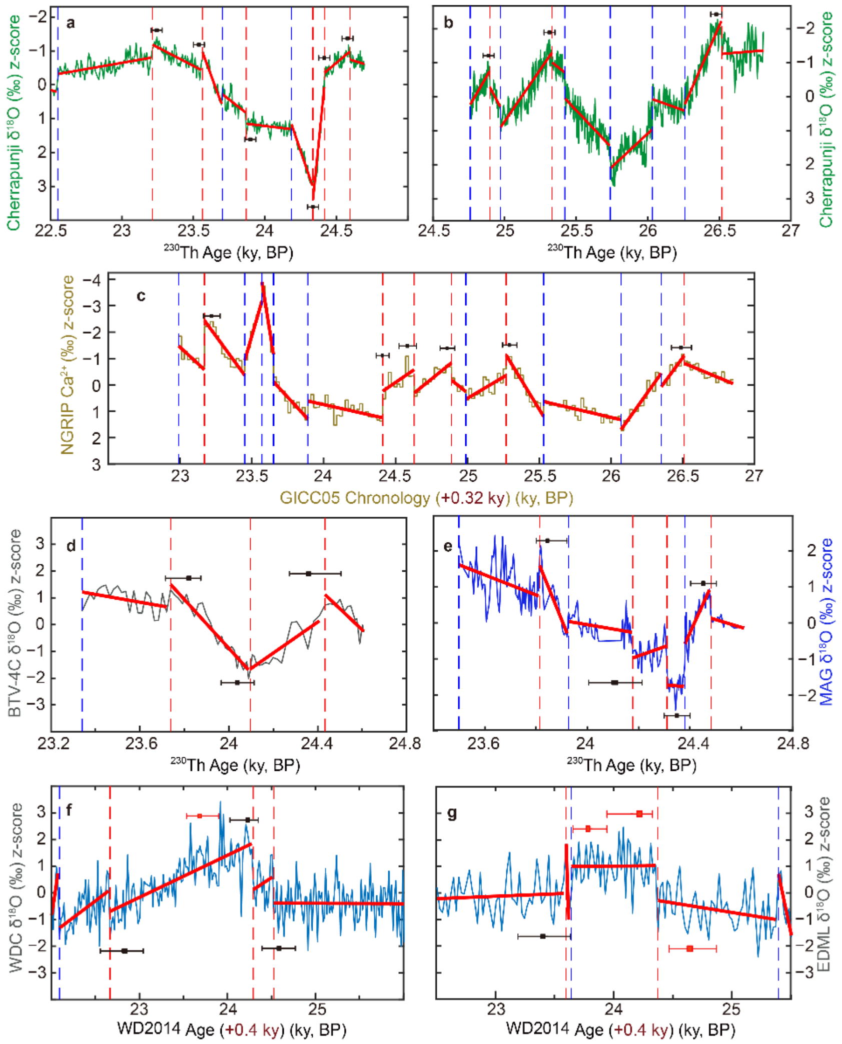

Supplementary Fig. 9 Trend-fitting results created by MATLAB. The red lines show the fitted trends. The vertical dashed lines are the change points picked by the "Trend-fitting" algorithm; the vertical red dashed lines are the key change points discussed in the main text. Error bars show the 2σ analytical error of the change points generated by the Ramp-fitting[63] and BREAKFIT[62] algorithms. We regarded the results as robust only in the case where the red dashed lines fall within the uncertainties of the associated error bars. The red error bars correspond to the change points that are not robust. The script of this method is provided in Supplementary Code 1, and the description of this method is shown in the Methods section. It is noted that we used the cropped MAG record, as same as in Supplementary Fig. 8c.

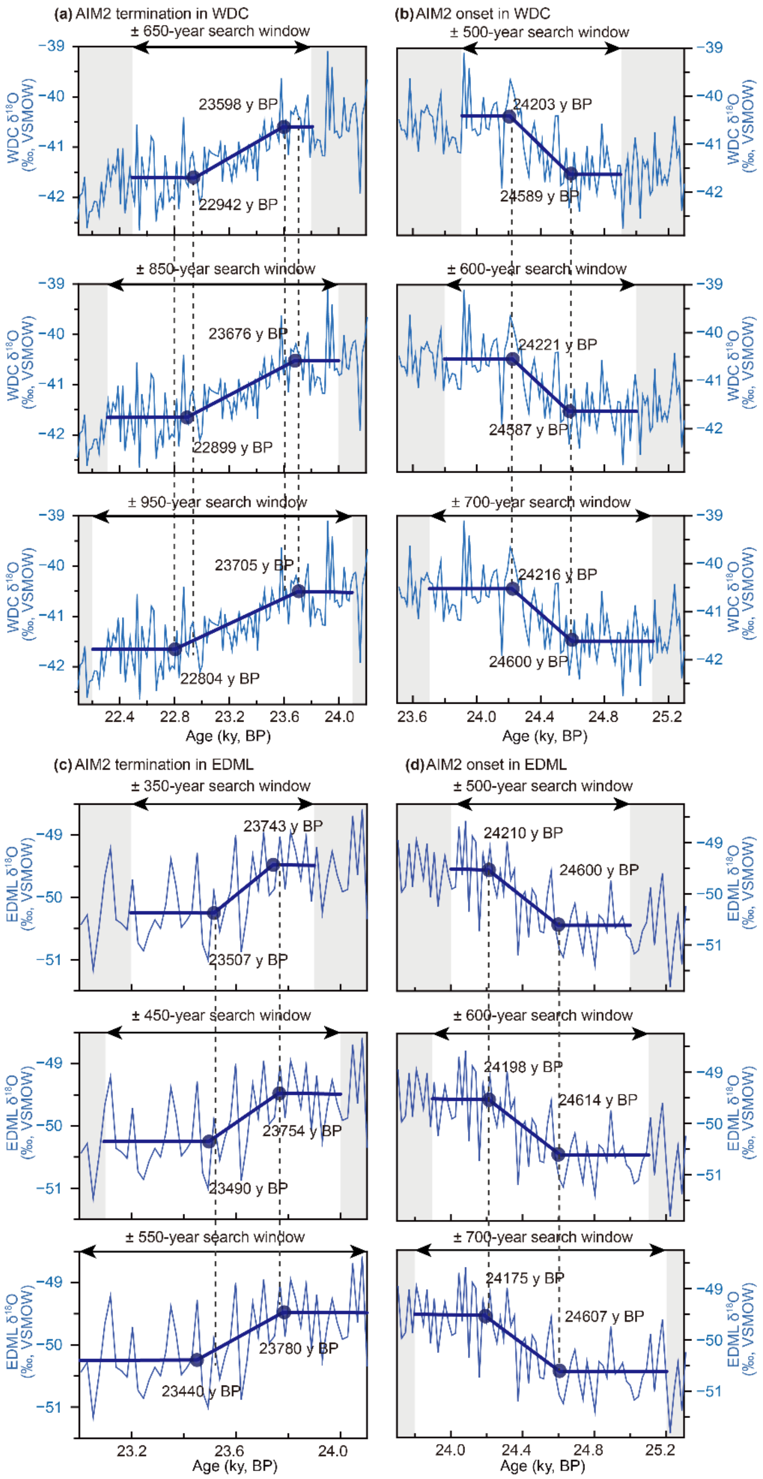

Supplementary Fig. 10 Sensitivity tests on the Antarctica ice-core $\delta^{18}O$ transitions during Antarctic Isotope Maximum 2 (AIM2). (**a**) AIM2 cooling transition in WDC ice-core. The bold blue ramps are defined by the Ramp-fitting algorithm[63] (see Methods) over three search windows of different widths. The change points are shown and the selected search window is indicated by the horizontal black arrows and the white boxes. (**b**)–(**d**) are the same as in (**a**) but for AIM2 warming transition in WDC, AIM2 cooling transition in EDML and AIM2 warming transition in EDML, respectively. The vertical dashed lines show the variation of change points when search time window changed, which is large (>60 years) in (**a**) and (**c**) thus they are regarded as not passing the sensitivity tests (see Methods).

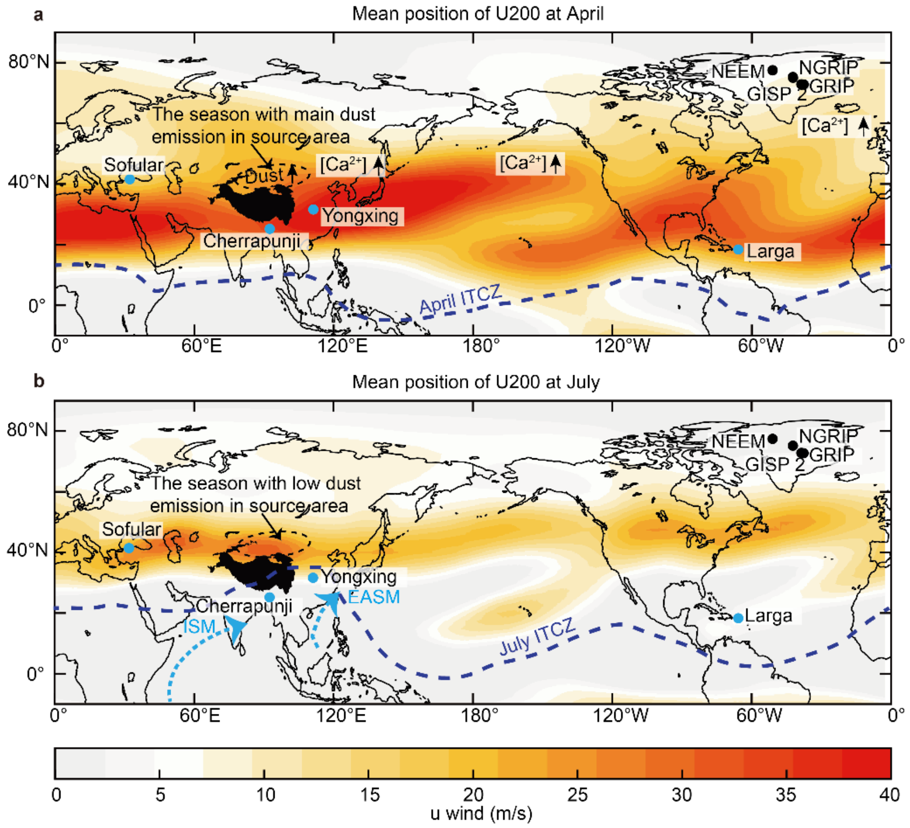

Supplementary Fig. 11 Modern upper tropospheric wind velocity in April and July. Background is the 200 hPa zonal (U) wind speed (in color scale) in (**a**) April, and (**b**) July, averaged for 1981–2010 CE. Monthly data are from the National Centers for Environmental Prediction–National Center for Atmospheric Research (NCEP-NCAR) reanalysis[86]. During the cold Heinrich stadials, the core axis of the westerly jet was located south of the Tibetan Plateau throughout most of the year, extending the dust season from spring to summer[56,87,88]. This enables more frequent dust emission and transportation to Greenland thus increasing the mineral-dust derived [Ca^{2+}]. The dotted ellipse depicts the approximate position of the Asian dust source regions (Taklimakan and Gobi deserts)[33]. The blue dots show the cave locations. The dotted blue arrows denote the low-level moisture trajectories of the ISM and the EASM. The black shading depicts the position of the Tibetan Plateau. Purple dashed curves show the modern position of the ITCZ in April (**a**) (approximately the average between January and July) and July (**b**), respectively[89]. ISM: Indian summer monsoon; EASM: East Asian summer monsoon. ITCZ: Intertropical Convergence Zone.

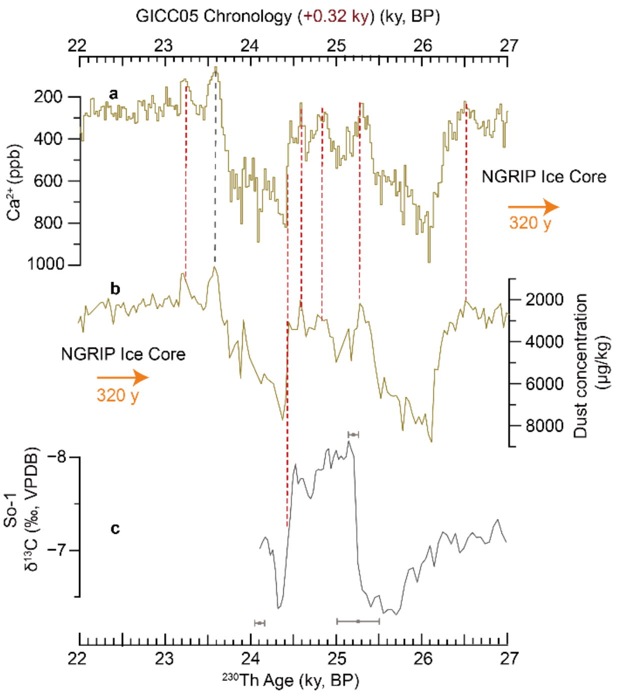

Supplementary Fig. 12 Comparison between the westerly-related records. (**a**) NGRIP ice-core [Ca^{2+}] record[20], (**b**) NGRIP ice-core dust record[90] and (**c**) Asian westerly domain Sofular Cave speleothem So-1 $\delta^{13}C$ record[91]. Ice-core records are plotted on the improved chronology (GICC05+320 years) via tuning Greenland [Ca^{2+}] time-series to the Cherrapunji $\delta^{18}O$ record (see main text). The dotted lines are the same as in Fig. 3. The gray error bars show ^{230}Th dates and their 2σ errors of the So-1 record. Note that the lack of ^{230}Th dating constraints prior to 25.3 ky BP in the So-1 record precludes a robust comparison between So-1 and Greenland records.

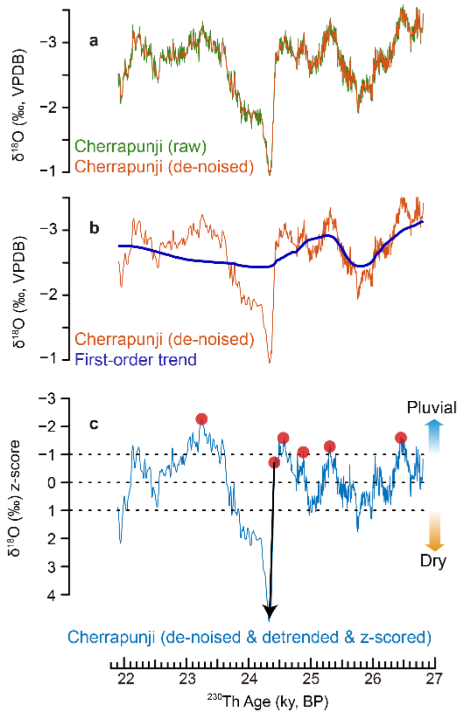

Supplementary Fig. 13 The denoised, detrended, and z-scored Cherrapunji Cave speleothem $\delta^{18}O$ record. (**a**) Raw $\delta^{18}O$ profile of Cherrapunji (green) and overlain by the de-noised $\delta^{18}O$ profile for Cherrapunji (orange). The white noise is removed by using the ensemble empirical mode decomposition function[92]. (**b**) The de-noised $\delta^{18}O$ profile for Cherrapunji record (orange) and overlain by the long-term first-order trend (dark blue). The long-term first-order trend is calculated using the singular spectrum analysis[93]. (**c**) De-noised, detrended and z-score transformed Cherrapunji record. Z-scored values delineate zero and number of decadal to multidecadal periods of inferred droughts (z-score > 1) and pluvial (z-score < −1) are highlighted by horizontal dotted black lines. The black arrow depicts the abrupt transition at the start of Asian Heinrich Period 2. The red dots correspond to the tie points used for tuning Greenland ice-core [Ca^{2+}] time series (see main text).

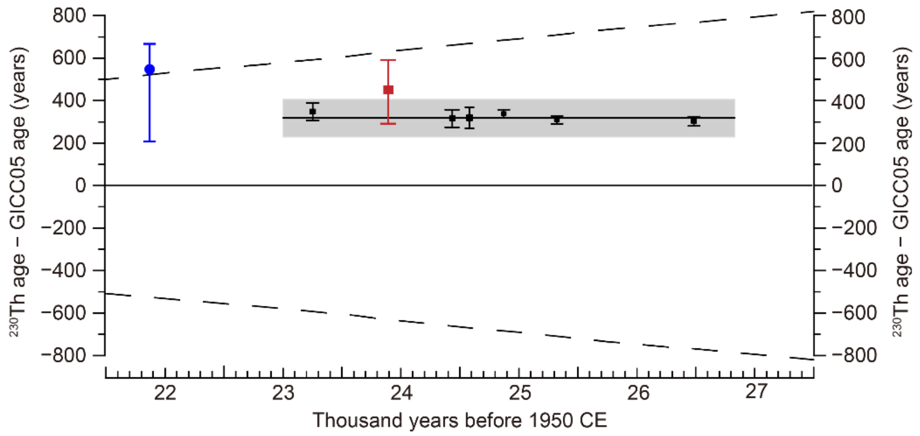

Supplementary Fig. 14 Timing differences of the tie points in speleothem ^{230}Th chronology and Greenland ice-core GICC05 chronology. The blue dot and error show the radionuclide tie-point and associated uncertainties[94]; The square and associated error bars show tie points and uncertainties in speleothems (red, ref. [42]; black, this study). The dashed lines indicate the maximum counting uncertainty of the GICC05 chronology[20]. The black line shows the improved Greenland chronology (GICC05+320 years) after synchronizing to speleothem Cherrapunji record using the six tie points, and the gray band depicts the 95.4% confidence interval. All errors are 2σ.

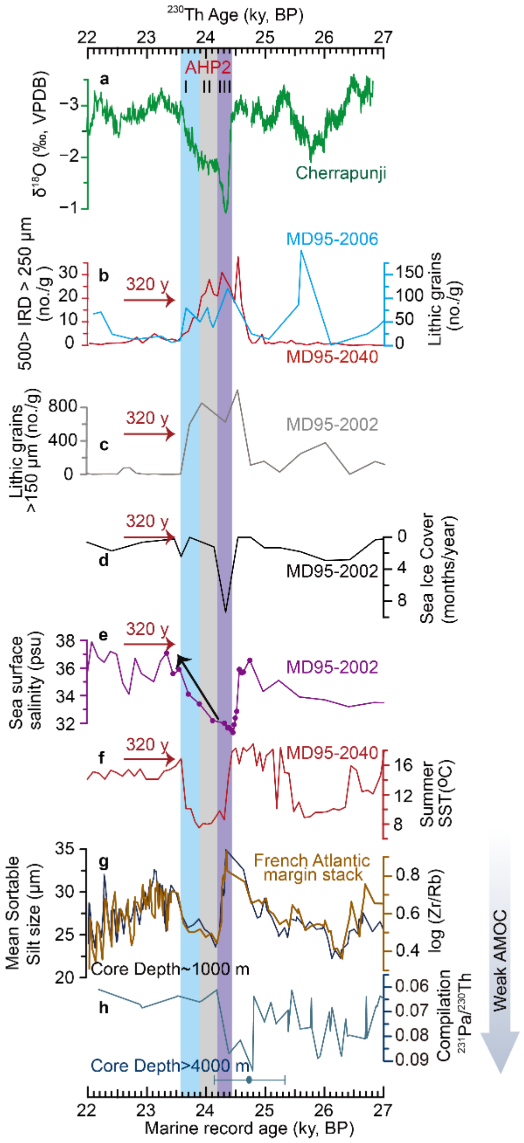

Supplementary Fig. 15 Comparison between Cherrapunji Cave record and marine sediment records. (**a**) Cherrapunji Cave speleothem $\delta^{18}O$ record (Cherrapunji record, this study). (**b**) Marine sediment records from MD95-2006[95] (light blue) and MD95-2040[96] cores (Maroon). (**c**)–(**e**) are marine sediment records from MD95-2002 core[97]. (**f**) Marine sediment record from MD95-2040 core[98]. (**b**)–(**f**) are plotted on the synchronized chronology consistent with GICC05[44] (Supplementary Note 1.8), which are shifted by +320 years. (**g**) Reconstructed glacial eastern boundary current based on cores from the French Atlantic margin[99]. (**h**) $^{231}Pa/^{230}Th$ from the Bermuda Rise (combination of ODP 1063[100] and CDH19[101]). Records in (**g**) and (**h**) reflect the strength of the AMOC[99,101]. Cave and core locations are shown in Supplementary Fig. 2. The black arrow depicts the increase of sea-surface salinity during the AHP2 termination. The vertical colored bars are the same as in Fig. 2. AHP2: Asian Heinrich Period 2; AMOC: Atlantic Meridional Overturning Circulation.

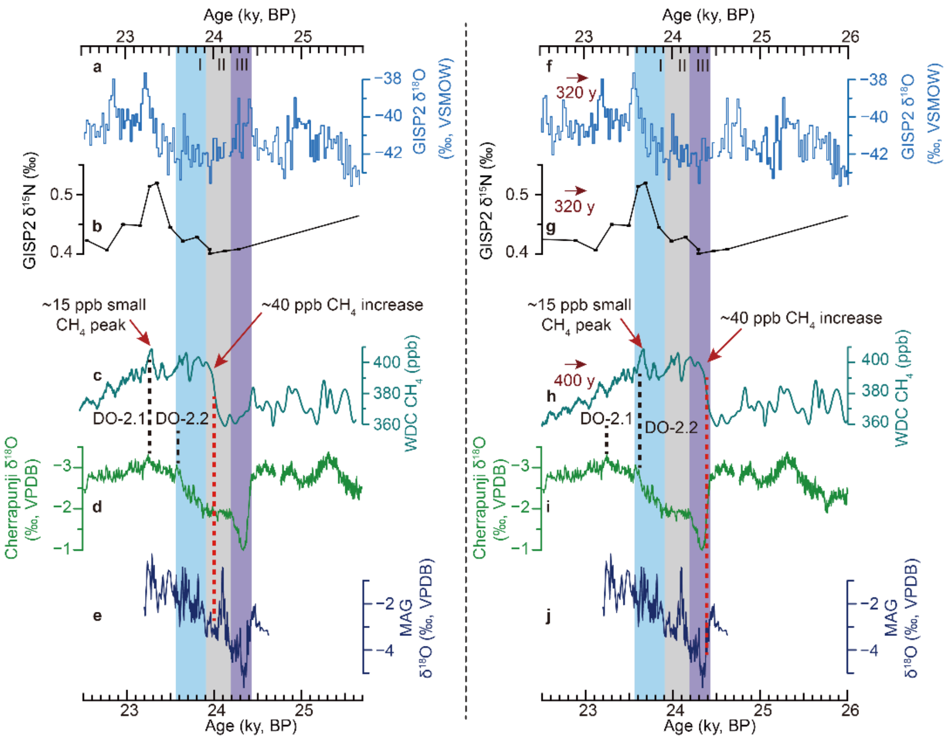

Supplementary Fig. 16 Comparison of the two correlation strategies between CH_4 and monsoon records. (**a**)–(**e**): Correlation strategy 1. (**a**) GISP2 $\delta^{18}O$ record[17,21], (**b**) GISP2 $\delta^{15}N$ record[20], (**c**) Antarctica WDC ice-core CH_4 record[65], (**d**) and (**e**) are speleothem $\delta^{18}O$ records from Cherrapunji and Marota caves (this study). Antarctic ice-core record is plotted on the WD2014 chronology[49]. Greenland ice-core records are plotted on the GICC05 gas and ice age scales[20,25-27]. The two vertical dashed black lines depict the DO-2.1 and DO-2.2 peaks. The vertical red dashed line corresponds to the ~40 ppb CH_4 increase and its correlation with monsoon records. (**f**)–(**j**): Correlation strategy 2 identified in this study. (**f**)–(**j**) are the same as in (**a**)–(**e**), but ice-core chronologies are on the improved chronologies proposed in this study. Vertical bars are the same as in Fig. 2. Cave and core locations are shown in Supplementary Fig. 2. AHP2: Asian Heinrich Period 2; SAHP2: South American Heinrich Period 2.

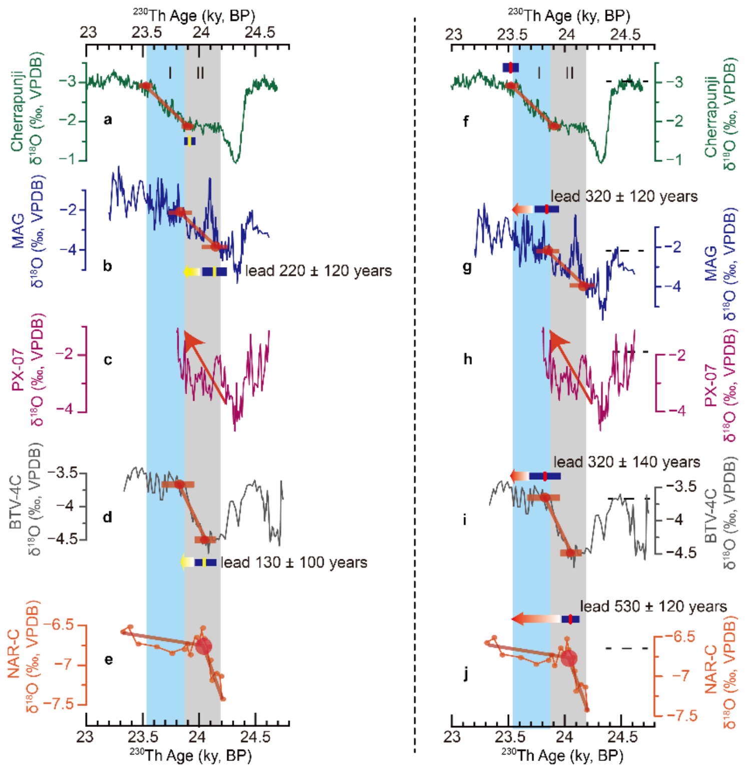

Supplementary Fig. 17 Comparison of change points in Asian summer monsoon and South American summer monsoon regions. (**a**)–(**e**): Cherrapunji, MAG, PX-07, BTV-4C and NAR-C $\delta^{18}O$ records. Blue horizontal bar with orange line in the middle represents change points and uncertainties (2σ) determined by calculating the combined uncertainty (see Methods). The records in (**f**)–(**j**) are the same as in (**a**)–(**e**). The error bars in (**f**)–(**j**) are the same as in (**a**)–(**e**) but for the end of AHP2/SAHP2. Horizontal dashed lines in (**f**)–(**j**) denote the pre-transition levels in different records. Red dots and associated error bars in (**a**)–(**j**) indicate timing and combined uncertainties (2σ) of the change points in speleothem $\delta^{18}O$ records (Supplementary Table 2), red lines show the fitted ramps and break lines generated by the Ramp-fitting[63] and BREAKFIT[62] algorithms, respectively (see Methods). The vertical colored bars are the same as in Fig. 2. AHP2: Asian Heinrich Period 2; SAHP2: South American Heinrich Period 2.

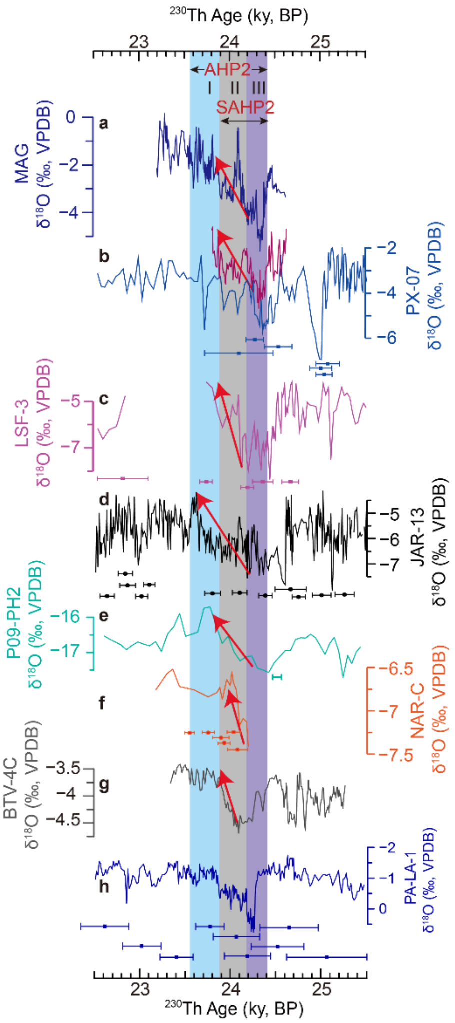

Supplementary Fig. 18 Speleothem records from South American and Central American summer monsoon regimes. (**a**)–(**g**) SASM speleothem δ^{18}O records from Marota Cave (MAG, this study), Paixão Cave (PX-07: magenta, this study; blue, ref. [78]), Lapa Sem Fim Cave (LSF-3[78]), Jaraguá Cave (JAR-13[102]), Pacupahuain Cave (P09-PH2[103]), Cueva del Diamante Cave (NAR-C, this study and ref. [104]) and Botuverá Cave (BTV-4C, this study), respectively. (**h**) Central American speleothem δ^{18}O record from Larga Cave[105] (Note the inverted y-axis). The red arrows show the long-term drying trend in the SASM regime. AHP2 and SAHP2 durations are marked by the double-sided arrows. Vertical bars are the same as in Fig. 2. Cave locations are shown in Supplementary Fig. 2. Error bars depict ^{230}Th dates and 2σ errors (color coded). SASM: South American summer monsoon.

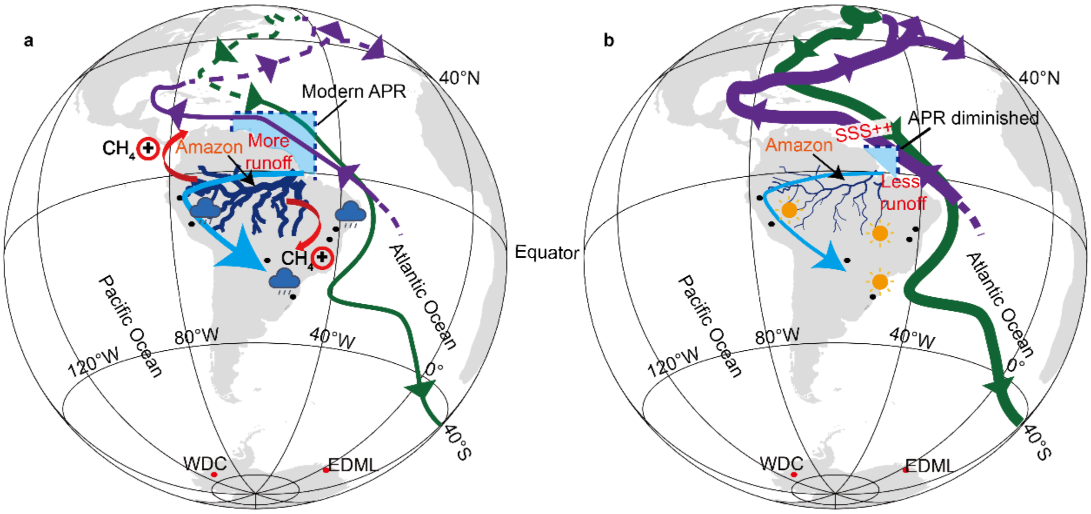

Supplementary Fig. 19 Conceptual diagram depicting the climatic dynamics during the SAHP2. (**a**) Schematic of hydroclimatic changes during the SAHP2 onset, including increased precipitation in the Southern Hemisphere tropical wetlands that produced excess atmospheric CH_4. (**a**) to (**b**) shows the centennial-scale changes in the Amazon River runoff prior to the AHP2 termination. The long-term drying trend in the SASM domain and associated reduction of the Amazon River runoff may have induced a positive sea-surface salinity (SSS) anomaly in the Amazon plume region (APR), which was subsequently advected to the North Atlantic[106,107]. These processes may have ultimately contributed to AMOC strengthening[108,109]. Black dots show locations of cave speleothem records, red dots show ice-core locations (Supplementary Fig. 2). Blue areas depict the modern APR (**a**) and the APR at the SAHP2 termination (**b**); the latter is arbitrary. The red arrows in (**a**) depict the emission of CH_4 to the atmosphere. The yellow sun symbol in (**b**) represents the drying condition in the SASM domain. The surface and deep currents are shown by the purple lines and green lines, respectively. The blue arrow indicates the low-level jet. The changes of Amazon river runoff and AMOC during the SAHP2 are consistent with the changes during the SAHP4[110]. The background figure was created using the M_Map software[111]. AHP2: Asian Heinrich Period 2; SAHP2(4): South American Heinrich Period 2(4); SASM: South American summer monsoon; AMOC: Atlantic meridional overturning circulation.

Dataset S1 (separate XLSX. file). ^{230}Th dating results for 9 speleothems.

Dataset S2 (separate XLSX. file). δ^{18}O time-series for 9 speleothem records, δ^{13}C time-series for speleothem Cherrapunji-2 and Cherrapunji-2017-1 and annual lamina thickness of speleothem Cherrapunji-2.

Dataset S3 (separate XLSX. file). Data of the main figures showed in the main text, including the data from referenced papers.

Supplementary Code (separate DOCX. file). Code used in the “Trend-fitting”.

Supplementary References

1 Stoll, H. *et al.* Interpretation of orbital scale variability in mid-latitude speleothem $\delta^{18}O$: Significance of growth rate controlled kinetic fractionation effects. *Quat. Sci. Rev.* **127**, 215–228 (2015). Doi: 10.1016/j.quascirev.2015.08.025.

3 Tan, L. et al. Rainfall variations in central Indo-Pacific over the past 2,700 y. *Proc. Natl. Acad. Sci. U. S. A.* **116**, 17201–17206 (2019). Doi:10.1073/pnas.1903167116.

3 Wang, Y. J. *et al.* A High-Resolution Absolute-Dated Late Pleistocene Monsoon Record from Hulu Cave, China. *Science* **294**, 2345–2348 (2001). Doi:10.1126/science.1064618.

4 Dong, X. *et al.* The termination period of Heinrich 2 event recorded by stalagmite in Indian monsoon domain [in Chinese]. *Quat. Sci.* **39**, 878–893 (2019). http://en.igg-journals.cn/article/doi/10.11928/j.issn.1001-7410.2019.04.08.

5 Caley, T., Roche, D. M. & Renssen, H. Orbital Asian summer monsoon dynamics revealed using an isotope-enabled global climate model. **5**, 5371 (2014). Doi:10.1038/ncomms6371.

6 Cheng, H. et al. Ice Age Terminations. *Science* **326**, 248–252 (2009). Doi:10.1126/science.1177840.

7 Pausata, F. S. R., Battisti, D. S., Nisancioglu, K. H. & Bitz, C. M. Chinese stalagmite $\delta^{18}O$ controlled by changes in the Indian monsoon during a simulated Heinrich event. *Nat. Geosci.* **4**, 474–480 (2011). Doi:10.1038/ngeo1169.

8 Yuan, D.X. et al. Timing, duration, and transitions of the last interglacial Asian monsoon. *Science* **5670** (2004). Doi: 10.1126/science.1091220.

9 Cheng, H. et al. The Asian monsoon over the past 640,000 years and ice age terminations. *Nature* **534**, 640–646 (2016).

10 Cheng, H. et al. Orbital-scale Asian summer monsoon variations: Paradox and exploration. *Sci. China-Earth Sci.* 64, 529–544 (2021). Doi:10.1007/s11430-020-9720-y.

11 Cheng, H. *et al.* Chinese stalagmite paleoclimate researches: A review and perspective. *Sci China Earth Sci* **62**, 1489–1513 (2019). Doi:10.1007/s11430-019-9478-3.

12 Kathayat, G. *et al.* Interannual oxygen isotope variability in Indian summer monsoon precipitation reflects changes in moisture sources. *Commun Earth Environ* **2**, 96 (2021). Doi:10.1038/s43247-021-00165-z.

13 Sun, Y. et al. A review of orbital-scale monsoon variability and dynamics in East Asia during the Quaternary. *Quat. Sci. Rev.* **288**, 107593 (2022). Doi:https://doi.org/10.1016/j.quascirev.2022.107593.

14 Zhao, J. et al. Reconstructing the western boundary variability of the Western Pacific Subtropical High over the past 200 years via Chinese cave oxygen isotope records. *Clim. Dyn.* **52**, 3741–3757 (2019). Doi:10.1007/s00382-018-4456-0.

15 Dorale, J. A. & Liu, Z. H. Limitations of Hendy Test criteria in judging the paleoclimatic suitability of speleothems and the need for replication. *J. Cave Karst Stud.* **71**, 73–80 (2009).

16 Gkinis, V. *et al.* A 120,000-year long climate record from a NW-Greenland deep ice core at ultra-high resolution. *Sci. Data* **8**, 141 (2021). Doi:10.1038/s41597-021-00916-9.

17 Grootes, P. M. & Stuiver, M. Oxygen 18/16 variability in Greenland snow and ice with 10^{-3}- to 10^{-5} year time resolution. *J. Geophys. Res.-Oceans* **102**, 26455–26470 (1997). Doi:10.1029/97JC00880.

18 Johnsen, S. J. *et al.* The $\delta^{18}O$ record along the Greenland Ice Core Project deep ice core and the

problem of possible Eemian climatic instability. *J. Geophys. Res.-Oceans* **102**, 26397-26410 (1997). Doi:10.1029/97JC0016.

19 North Greenland Ice Core Project Members. High-resolution record of Northern Hemisphere climate extending into the last interglacial period. *Nature* **431**, 147–151 (2004). Doi:10.1038/nature02805.

20 Rasmussen, S. O. *et al.* A stratigraphic framework for abrupt climatic changes during the Last Glacial period based on three synchronized Greenland ice-core records: refining and extending the INTIMATE event stratigraphy. *Quat. Sci. Rev.* **106**, 14–28 (2014). Doi: 10.1016/j.quascirev.2014.09.007.

21 Stuiver, M. & Grootes, P.M.. GISP2 oxygen isotope ratios. *Quat. Res.* **53**, 277e284 (2000). Doi: 10.1006/qres.2000.2127.

22 Fuhrer, K., Neftel, A., Anklin, M. & Maggi, V. Continuous measurements of hydrogen peroxide, formaldehyde, calcium and ammonium concentrations along the new grip ice core from summit, Central Greenland. *Atmospheric Environment. Part A. General Topics* **27**, 1873–1880 (1993). Doi: 10.1016/0960-1686(93)90292-7.

23 Mayewski, P. A. *et al.* Major features and forcing of high-latitude northern hemisphere atmospheric circulation using a 110,000-year-long glaciochemical series. *J. Geophys. Res.-Oceans* **102**, 26345–26366 (1997). Doi: 10.1029/96JC03365.

24 Schüpbach, S. *et al.* Greenland records of aerosol source and atmospheric lifetime changes from the Eemian to the Holocene. *Nat. Commun.* **9**, 1476, doi:10.1038/s41467-018-03924-3 (2018).

25 Andersen, K. K. *et al.* The Greenland Ice Core Chronology 2005, 15–42 ka. Part 1: constructing the time scale. *Quat. Sci. Rev.* **25**, 3246–3257 (2006). Doi: 10.1016/j.quascirev.2006.08.002.

26 Rasmussen, S. O. *et al.* Synchronization of the NGRIP, GRIP, and GISP2 ice cores across MIS 2 and palaeoclimatic implications. *Quat. Sci. Rev.* **27**, 18–28 (2008). Doi: 10.1016/j.quascirev.2007.01.016.

27 Seierstad, I. K. *et al.* Consistently dated records from the Greenland GRIP, GISP2 and NGRIP ice cores for the past 104 ka reveal regional millennial-scale $\delta^{18}O$ gradients with possible Heinrich event imprint. *Quat. Sci. Rev.* **106**, 29–46 (2014). Doi: 10.1016/j.quascirev.2014.10.032.

28 Steffensen, J. P. The size distribution of microparticles from selected segments of the Greenland Ice Core Project ice core representing different climatic periods. *J. Geophys. Res.-Oceans* **102**, 26755-26763 (1997). Doi: 10.1029/97JC01490.

29 Biscaye, P. E. *et al.* Asian provenance of glacial dust (stage 2) in the Greenland Ice Sheet Project 2 Ice Core, Summit, Greenland. *J. Geophys. Res.-Oceans* **102**, 26765–26781 (1997). Doi: 10.1029/97JC01249.

30 Bory, A. J. M., Biscaye, P. E. & Grousset, F. E. Two distinct seasonal Asian source regions for mineral dust deposited in Greenland (NorthGRIP). *Geophys. Res. Lett.* **30** (2003). Doi: 10.1029/2002GL016446.

31 Svensson, A., Biscaye, P. E. & Grousset, F. E. Characterization of late glacial continental dust in the Greenland Ice Core Project ice core. *J. Geophys. Res.-Atmos.* **105**, 4637–4656, (2000). Doi: 10.1029/1999JD901093.

32 Újvári, G. *et al.* Two possible source regions for central Greenland last glacial dust. *Geophys. Res. Lett.* **42**, 10,399–10,408 (2015). Doi: 10.1002/2015GL066153.

33 Sun, Y. B. *et al.* Source-to-sink fluctuations of Asian aeolian deposits since the late Oligocene. *Earth-Sci. Rev.* **200**, 102963 (2020).

34 Han, C. *et al.* High-resolution isotopic evidence for a potential Saharan provenance of Greenland glacial dust. *Sci Rep* **8**, 15582 (2018). Doi:10.1038/s41598-018-33859-0.

35 Uno, I. *et al.* Asian dust transported one full circuit around the globe. *Nat. Geosci.* **2**, 557–560 (2009). Doi:10.1038/ngeo583.

36 Bory, A. J. M., Biscaye, P. E., Svensson, A. & Grousset, F. E. Seasonal variability in the origin of recent atmospheric mineral dust at NorthGRIP, Greenland. *Earth Planet. Sci. Lett.* **196**, 123–134 (2002). Doi: 10.1016/S0012-821X(01)00609-4.

37 Kang, J.-H. *et al.* Mineral dust and major ion concentrations in snowpit samples from the NEEM site, Greenland. *Atmos. Environ.* **120**, 137–143 (2015). Doi: 10.1016/j.atmosenv.2015.08.062.

38 Sun, J. & Zhao, L. Numerical simulation of two East Asian dust storms in spring 2006. *Earth Surf. Process. Landf.* **33**, 1892–1911 (2008). Doi: 10.1002/esp.1734.

39 Yuan, G. Characteristics and Cause of the Sandstorm in Inner Mongolia in 2001-2015 [in Chinese]. *Journal of desert research* **37**, 1204–1209 (2017). https://oversea.cnki.net/kcms/detail/detail.aspx?filename=ZGSS201706020&dbcode=CJFD&dbname=CJFD2017&v=

40 An, Z. S. *et al.* Interplay between the Westerlies and Asian monsoon recorded in Lake Qinghai sediments since 32 ka. *Sci Rep* **2**, 619 (2012). Doi:10.1038/srep00619.

41 Fischer, H., Siggaard-Andersen, M.-L., Ruth, U., Röthlisberger, R. & Wolff, E. Glacial/interglacial changes in mineral dust and sea-salt records in polar ice cores: Sources, transport, and deposition. *Rev. Geophys.* **45** (2007). Doi: 10.1029/2005RG000192.

42 Duan, F. *et al.* A 3000-yr annually laminated stalagmite record of the Last Glacial Maximum from Hulu Cave, China. *Quat. Res.* **83**, 360–369 (2015). Doi: 10.1016/j.yqres.2015.01.003.

43 Partin, J. W., Cobb, K. M., Adkins, J. F., Clark, B. & Fernandez, D. P. Millennial-scale trends in west Pacific warm pool hydrology since the Last Glacial Maximum. *Nature* **449**, 452–455 (2007). Doi:10.1038/nature06164.

44 Waelbroeck, C. *et al.* Consistently dated Atlantic sediment cores over the last 40 thousand years. *Sci. Data* **6**, 165 (2019). Doi:10.1038/s41597-019-0173-8.

45 WAIS Divide Project Members. Precise interpolar phasing of abrupt climate change during the last ice age. *Nature* **520**, 661–665 (2015).

46 EPICA Community Members. One-to-one coupling of glacial climate variability in Greenland and Antarctica. *Nature* **444**, 195–198 (2006). Doi:10.1038/nature05301.

47 Buizert, C. *et al.* Abrupt ice-age shifts in southern westerly winds and Antarctic climate forced from the north. *Nature* **563**, 681–685 (2018). Doi:10.1038/s41586-018-0727-5.

48 Markle, B. R., Steig, E. J., Roe, G. H., Winckler, G. & McConnell, J. R. Concomitant variability in high-latitude aerosols, water isotopes and the hydrologic cycle. *Nat. Geosci.* **11**, 853–859 (2018). Doi:10.1038/s41561-018-0210-9.

49 Sigl, M. *et al.* The WAIS Divide deep ice core WD2014 chronology – Part 2: Annual-layer counting (0–31 ka BP). *Clim. Past* **12**, 769–786 (2016). Doi:10.5194/cpd-11-3425-2015.

50 Markle, B. R. *et al.* Global atmospheric teleconnections during Dansgaard–Oeschger events. *Nat. Geosci.* **10**, 36–40 (2017). Doi:10.1038/ngeo2848.

51 Lambert, F., Bigler, M., Steffensen, J. P., Hutterli, M. & Fischer, H. Centennial mineral dust variability in high-resolution ice core data from Dome C, Antarctica. *Clim. Past* **8**, 609–623 (2012).

Doi:10.5194/cp-8-609-2012.

52 Schüpbach, S. *et al.* High-resolution mineral dust and sea ice proxy records from the Talos Dome ice core. *Clim. Past* **9**, 2789–2807 (2013). Doi:10.5194/cp-9-2789-2013.

53 Albani, S., Mahowald, N. M., Delmonte, B., Maggi, V. & Winckler, G. Comparing modeled and observed changes in mineral dust transport and deposition to Antarctica between the Last Glacial Maximum and current climates. *Clim. Dyn.* **38**, 1731–1755 (2012). Doi:10.1007/s00382-011-1139-5.

54 Delmonte, B. *et al.* Aeolian dust in East Antarctica (EPICA-Dome C and Vostok): Provenance during glacial ages over the last 800 kyr. *Geophys. Res. Lett.* **35** (2008). Doi: 10.1029/2008GL033382.

55 Pinho, T. M. L. *et al.* Meridional changes in the South Atlantic Subtropical Gyre during Heinrich Stadials. *Sci Rep* **11**, 9419 (2021). Doi:10.1038/s41598-021-88817-0.

56 Chiang, J. C. H. *et al.* Role of seasonal transitions and westerly jets in East Asian paleoclimate. *Quat. Sci. Rev.* **108**, 111–129 (2015). Doi: 10.1016/j.quascirev.2014.11.009.

57 Chiang, J. C. H., Swenson, L. M. & Kong, W. Role of seasonal transitions and the westerlies in the interannual variability of the East Asian summer monsoon precipitation. *Geophys. Res. Lett.* **44**, 3788–3795 (2017). Doi: 10.1002/2017GL072739.

58 Kong, W., Swenson, L. M. & Chiang, J. C. H. Seasonal Transitions and the Westerly Jet in the Holocene East Asian Summer Monsoon. *J. Clim.* **30**, 3343–3365 (2017). Doi:10.1175/JCLI-D-16-0087.1.

59 Zhang, H. B. *et al.* East Asian hydroclimate modulated by the position of the westerlies during Termination I. *Science* **362**, 580–583 (2018). Doi:10.1126/science.aat9393.

60 Cai, Y. J. *et al.* Variability of stalagmite-inferred Indian monsoon precipitation over the past 252,000 y. *Proc. Natl. Acad. Sci. U. S. A.* **112**, 2954 (2015). Doi:10.1073/pnas.1424035112.

61 Chiang, J. C. H., Herman, M. J., Yoshimura, K. & Fung, I. Y. Enriched East Asian oxygen isotope of precipitation indicates reduced summer seasonality in regional climate and westerlies. *Proc. Natl. Acad. Sci. U. S. A.* **117**, 14745 (2020). Doi:10.1073/pnas.1922602117.

62 Mudelsee, M. Break function regression. *Eur. Phys. J.-Spec. Top.* **174**, 49-63 (2009). Doi:10.1140/epjst/e2009-01089-3.

63 Capron, E. *et al.* The anatomy of past abrupt warmings recorded in Greenland ice. *Nat. Commun.* **12**, 2106 (2021). Doi:10.1038/s41467-021-22241-w.

64 Cheng, H. *et al.* Atmospheric $^{14}C/^{12}C$ changes during the last glacial period from Hulu Cave. *Science* **362**, 1293–1297 (2018).

65 Rhodes, R. H. *et al.* Enhanced tropical methane production in response to iceberg discharge in the North Atlantic. *Science* **348**, 1016 (2015). Doi:10.1126/science.1262005.

66 Bauska, T. K., Marcott, S. A. & Brook, E. J. Abrupt changes in the global carbon cycle during the last glacial period. *Nat. Geosci.* **14**, 91–96 (2021). Doi:10.1038/s41561-020-00680-2.

67 Spratt, R. M. & Lisiecki, L. E. A Late Pleistocene sea level stack. *Clim. Past* **12**, 1079-1092 (2016). Doi:10.5194/cp-12-1079-2016.

68 Buizert, C. *et al.* The WAIS Divide deep ice core WD2014 chronology – Part 1: Methane synchronization (68–31 ka BP) and the gas age–ice age difference. *Clim. Past* **11**, 153–173 (2015). Doi:10.5194/cp-11-153-2015.

69 Clark, P. U. *et al.* The Last Glacial Maximum. *Science* **325**, 710–714 (2009).

70 Zhang, Y. *et al.* Different precipitation patterns across tropical South America during Heinrich and

Dansgaard-Oeschger stadials. *Quat. Sci. Rev.* **177**, 1-9 (2017). Doi: 10.1016/j.quascirev.2017.10.012.

71 Campos, M. C. *et al.* A new mechanism for millennial scale positive precipitation anomalies over tropical South America. *Quat. Sci. Rev.* **225**, 105990 (2019). Doi: 10.1016/j.quascirev.2019.105990.

72 Jaeschke, A., Rühlemann, C., Arz, H., Heil, G. & Lohmann, G. Coupling of millennial-scale changes in sea surface temperature and precipitation off northeastern Brazil with high-latitude climate shifts during the last glacial period. *Paleoceanography* **22** (2007). Doi: 10.1029/2006PA001391.

73 Deplazes, G. *et al.* Links between tropical rainfall and North Atlantic climate during the last glacial period. *Nat. Geosci.* **6**, 213–217 (2013). Doi:10.1038/ngeo1712.

74 Breitenbach, S. F. M. *et al.* COnstructing Proxy Records from Age models (COPRA). *Clim. Past* **8**, 1765–1779 (2012). Doi:10.5194/cp-8-1765-2012.

75 Ramsey, C. B. & Lee, S. Recent and Planned Developments of the Program OxCal. *Radiocarbon* **55**, 720–730 (2013). Doi:10.1017/S0033822200057878.

76 Scholz, D. & Hoffmann, D. L. StalAge – An algorithm designed for construction of speleothem age models. *Quat. Geochronol.* **6**, 369–382 (2011).

77 Chen, S. T. *et al.* Strong coupling of Asian Monsoon and Antarctic climates on sub-orbital timescales. *Sci Rep* **6**, 32995 (2016). Doi:10.1038/srep32995.

78 Stríkis, N. M. *et al.* South American monsoon response to iceberg discharge in the North Atlantic. *Quat. Sci. Rev.* **115**, 3788 (2018). Doi:10.1073/pnas.1717784115.

79 Wang, X. *et al.* Interhemispheric anti-phasing of rainfall during the last glacial period. *Quat. Sci. Rev.* **25**, 3391–3403 (2006). Doi:https://doi.org/10.1016/j.quascirev.2006.02.009.

80 Chen, S.T., Wang, Y.J., Wu, J.Y. & Liu, D.B. An event of the East Asian monsoon responding to Heinrich Event 2: Evidence from high-resolution stalagmite record [in Chinese]. *Geochimica* **35**, 586–592 (2006).

81 Liu, D. B. *et al.* Contrasting Patterns in Abrupt Asian Summer Monsoon Changes in the Last Glacial Period and the Holocene. *Paleoceanogr. Paleoclimatology* **33**, 214–226 (2018). Doi:https://doi.org/10.1002/2017PA003294.

82 Li, T.-Y. *et al.* High precise dating on the variation of the Asian summer monsoon since 37 ka BP. *Sci Rep* **11**, 9375 (2021). Doi:10.1038/s41598-021-88597-7.

83 Zhao, K., Wang, Y. J., Edwards, R. L., Cheng, H. & Liu, D. High-resolution stalagmite $\delta^{18}O$ records of Asian monsoon changes in central and southern China spanning the MIS 3/2 transition. *Earth Planet. Sci. Lett.* **298**, 191–198 (2010). Doi:https://doi.org/10.1016/j.epsl.2010.07.041.

84 Becker, A., Finger, P., Meyer–Christoffer, A., Rudolf, B. & Ziese, M. GPCC Full Data Reanalysis Version 6.0 at 1.0: Monthly Land-Surface Precipitation from RainGauges Built on GTS–Based and Historic Data. Global Precipitation Climatology Centre (GPCC), Berlin, Germany. (2011). DOI: 10.5065/D6000072.

85 Rienecker, M. M. *et al.* MERRA: NASA's Modern-Era Retrospective Analysis for Research and Applications. *J. Clim*. **24**, 3624–3648 (2011). Doi:10.1175/JCLI-D-11-00015.1.

86 Kalnay, E. *et al.* The NCEP/NCAR 40-Year Reanalysis Project. *Bull. Amer. Meteor. Soc.* **77**, 437–472 (1996).

87 Nagashima, K. *et al.* Millennial-scale oscillations of the westerly jet path during the last glacial period. *J. Asian Earth Sci.* **40**, 1214–1220 (2011). Doi: 10.1016/j.jseaes.2010.08.010.

88 Serno, S. *et al.* Change in dust seasonality as the primary driver for orbital-scale dust storm variability

in East Asia. *Geophys. Res. Lett.* **44**, 3796–3805 (2017). Doi: 10.1002/2016GL072345.

89 Cheng, H., Sinha, A., Wang, X. F., Cruz, F. & Edwards, R. The Global Paleomonsoon as seen through speleothem records from Asia and the Americas. *Clim. Dyn.* **39**, 1045–1062, doi:10.1007/s00382-012-1363-7 (2012).

90 Ruth, U. *et al.* Ice core evidence for a very tight link between North Atlantic and east Asian glacial climate. *Geophys. Res. Lett.* **34** (2007). Doi: 10.1029/2006GL027876.

91 Fleitmann, D. *et al.* Timing and climatic impact of Greenland interstadials recorded in stalagmites from northern Turkey. *Geophys. Res. Lett.* **36** (2009). Doi:https://doi.org/10.1029/2009GL040050.

92 Wu, Z. & Huang, N. E. Ensemble empirical mode decomposition: a noise-assisted data analysis method. *Adv Adapt Data Anal* **01**, 1–41 (2009). Doi:10.1142/S1793536909000047.

93 Ghil, M. *et al.* Advanced spectral methods for climatic time series. *Rev. Geophys.* **40**, 3–41 (2002). Doi:https://doi.org/10.1029/2000RG000092.

94 Adolphi, F. *et al.* Connecting the Greenland ice-core and U/Th timescales via cosmogenic radionuclides: testing the synchroneity of Dansgaard–Oeschger events. *Clim. Past* **14**, 1755–1781 (2018). Doi:10.5194/cp-14-1755-2018.

95 Knutz, P. C., Austin, W. E. N. & Jones, E. J. W. Millennial-scale depositional cycles related to British Ice Sheet variability and North Atlantic paleocirculation since 45 kyr B.P., Barra Fan, U.K. margin. *Paleoceanography* **16**, 53–64, (2001). Doi: 10.1029/1999PA000483.

96 Schönfeld, J., Zahn, R. & de Abreu, L. Surface and deep water response to rapid climate changes at the Western Iberian Margin. *Glob. Planet. Change* **36**, 237–264 (2003). Doi: 10.1016/S0921-8181(02)00197-2.

97 Eynaud, F. *et al.* New constraints on European glacial freshwater releases to the North Atlantic Ocean. *Geophys. Res. Lett.* **39** (2012). Doi: 10.1029/2012GL052100.

98 Salgueiro, E. et al. Temperature and productivity changes off the western Iberian margin during the last 150 ky. *Quat. Sci. Rev.* **29**, 680–695 (2010). Doi: 10.1016/j.quascirev.2009.11.013.

99 Toucanne, S. *et al.* The North Atlantic Glacial Eastern Boundary Current as a Key Driver for Ice Sheet—AMOC Interactions and Climate Instability. *Paleoceanogr. Paleoclimatolog* **36**, e2020PA004068 (2021). Doi:https://doi.org/10.1029/2020PA004068.

100 Böhm, E. *et al.* Strong and deep Atlantic meridional overturning circulation during the last glacial cycle. *Nature* **517**, 73–76 (2015). Doi:10.1038/nature14059.

101 Henry, L. G. *et al.* North Atlantic ocean circulation and abrupt climate change during the last glaciation. *Science* **353** (2016).

102 Novello, V. F. *et al.* A high-resolution history of the South American Monsoon from Last Glacial Maximum to the Holocene. *Sci Rep* **7**, 44267 (2017). Doi:10.1038/srep44267.

103 Kanner, L. C., Burns, S. J., Cheng, H. & Edwards, R. L. High-Latitude Forcing of the South American Summer Monsoon During the Last Glacial. *Science* **335**, 570 (2012). Doi:10.1126/science.1213397.

104 Cheng, H. *et al.* Climate change patterns in Amazonia and biodiversity. *Nat. Commun.* **4**, 1411 (2013). Doi:10.1038/ncomms2415.

105 Warken, S. F. *et al.* Persistent Link Between Caribbean Precipitation and Atlantic Ocean Circulation During the Last Glacial Revealed by a Speleothem Record From Puerto Rico. *Paleoceanogr. Paleoclimatology* **35**, e2020PA003944 (2020). Doi:https://doi.org/10.1029/2020PA003944.

106 Broecker, W. S. *The Great Ocean Conveyor* (Princeton Univ. Press, 1991).

107 Burckel, P. *et al.* Changes in the geometry and strength of the Atlantic meridional overturning circulation during the last glacial (20–50 ka). *Clim. Past* **12**, 2061–2075 (2016). Doi:10.5194/cp-12-2061-2016.

108 Jahfer, S., Vinayachandran, P. N. & Nanjundiah, R. S. The role of Amazon river runoff on the multidecadal variability of the Atlantic ITCZ. *Environ. Res. Lett.* **15**, 054013 (2020). Doi:10.1088/1748-9326/ab7c8a.

109 Krebs, U. & Timmermann, A. Tropical Air–Sea Interactions Accelerate the Recovery of the Atlantic Meridional Overturning Circulation after a Major Shutdown. *J. Clim.* **20**, 4940–4956 (2007). Doi:10.1175/JCLI4296.1.

110 Cheng, H. *et al.* Onset and termination of Heinrich Stadial 4 and the Underlying Climate Dynamics. *Commun Earth Environ* **2**, 230 (2021). DOI:10.1038/s43247-021-00304-6.

111 Pawlowicz, R.. M_Map: A mapping package for MATLAB. Version 1.4m, [Computer software]. www.eoas.ubc.ca/~rich/map.html (2020).
