# Supplementary material for: Coupled atmosphere-ice-ocean dynamics during Heinrich Stadial 2
Source: Nat Commun. 2022 Oct 4;13:5867. doi: 10.1038/s41467-022-33583-4 (PMC9532435; doi:10.1038/s41467-022-33583-4)
Supplement: Supplementary file 3 — Description of Additional Supplementary Files [file 41467_2022_33583_MOESM3_ESM.pdf]

Supplementary Data 1: 230Th dating results for 9 speleothems.

Supplementary Data 2: δ 18O time-series for 9 speleothem records, δ 13C time-series for speleothem Cherrapunji-2 and Cherrapunji-2017-1 and annual lamina counting result for speleothem Cherrapunji-2.

Supplementary Data 3: Data of the main figures showed in the main text, including the data from referenced papers.

Supplementary Code 1: Code used in the "Trend-fitting".
