# Supplementary material for: Coupled atmosphere-ice-ocean dynamics during Heinrich Stadial 2
Source: Nat Commun. 2022 Oct 4;13:5867. doi: 10.1038/s41467-022-33583-4 (PMC9532435; doi:10.1038/s41467-022-33583-4)
Supplement: Supplementary file 7 — Supplementary Code 1 [file 41467_2022_33583_MOESM7_ESM.docx]

% This collection of MATLAB code is a supplement to the paper:

%

% Dong et al., Coupled atmosphere-ice-ocean dynamics during Heinrich Stadial 2

% (2022)

% The code performs the “trend-fitting” analysis

% as described in that work.

%

% To run the code, the user should put the name of .csv file in Line28,

% The file should include two columns (first: age; Second: proxy record) with no title.

% The .csv file should be put in the same folder of this code

% Then the user can set the maximum number of trending lines in Line33

% In case of questions or comments, please contact me on the address below.

% Thanks for your interest in this work.

%

% Kind regards,

%

% Xiyu Dong

%

% Email: baoweie0@stu.xjtu.edu.cn

% Institute of Global Environmental Change, Xi'an Jiaotong University, Xi’an, 710049, China

% Detect changes in trend

clear all; clc; clf;

data = load ('proxy.csv'); % **the user should put the name of .csv file here**

t=data(:,1);

x=data(:,2);

x=zscore(x);

cp_max=4; %% **the user can set Maximum number of trending lines**

%% replot, age vs variables

id=findchangepts(x,'MaxNumChanges',cp_max,'Statistic','linear');

id=[id;id-1];

id=sort(id);

id=[1, id', length(x)];% get all the transition positions and start and end points

k=1;

figure(1)

plot(t,x); hold on

for i=1:2:length(id)

N=length(t(id(i):id(i+1))); % get the length of each interval

T=[t(id(i):id(i+1)) ones(N,1)]; % for regression function's request: generate a vector of one beside to get weight and intercept

A(:,k)=regress(x(id(i):id(i+1)),T);

plot(t(id(i):id(i+1)),t(id(i):id(i+1))*A(1,k)+A(2,k),'r','linewidth',2);%plot the linear trend

hold on;

line([t(id(i)) t(id(i))],[-4 4],'LineWidth',0.75,'Color','blue','LineStyle','--');% plot the seperate line

hold on;

k=k+1;

end

set(gca,'Ydir','reverse');
